# Genome-scale CRISPR knockout screen identifies *TIGAR* as a modifier of PARP inhibitor sensitivity

**DOI:** 10.1038/s42003-019-0580-6

**Published:** 2019-09-09

**Authors:** Pingping Fang, Cristabelle De Souza, Kay Minn, Jeremy Chien

**Affiliations:** 1 Department of Pharmacology, Toxicology and Therapeutics, The University of Kansas Medical Center, Kansas City, KS 66160 USA; 2 Department of Internal Medicine, University of New Mexico Cancer Center, University of New Mexico School of Medicine, Albuquerque, NM 87131 USA; 3 Department of Biochemistry and Molecular Medicine, University of California, Davis, Sacramento, CA 95816 USA; 4 Department of Pathology and Laboratory Medicine, The University of Kansas Medical Center, Kansas City, KS 66160 USA; 5 Department of Obstetrics and Gynecology, University of California, Davis, Sacramento, CA 95816 USA

**Keywords:** Cancer metabolism, Targeted therapies, Ovarian cancer, Cancer genomics

## Abstract

Treatment of cancer with poly (ADP-ribose) polymerase (PARP) inhibitors is currently limited to cells defective in the homologous recombination (HR) pathway. Identification of genetic targets that induce or mimic HR deficiencies will extend the clinical utility of PARP inhibitors. Here we perform a CRISPR/Cas9-based genome-scale loss-of-function screen, using the sensitivity of PARP inhibitor olaparib as a surrogate. We identify *C12orf5*, encoding TP53 induced glycolysis and apoptosis regulator (TIGAR), as a modifier of PARP inhibitor response. We show that TIGAR is amplified in several cancer types, and higher expression of TIGAR associates with poor overall survival in ovarian cancer. TIGAR knockdown enhances sensitivity to olaparib in cancer cells via downregulation of BRCA1 and the Fanconi anemia pathway and increases senescence of these cells by affecting metabolic pathways and increasing the cytotoxic effects of olaparib. Our results indicate TIGAR should be explored as a therapeutic target for treating cancer and extending the use of PARP inhibitors.

## Abstract (web summary)

Pingping Fang et al. report a CRISPR/Cas9-based genome-scale loss-of-function screen identifying TIGAR as a modifier of response to PARP inhibition. The authors find that knockdown of TIGAR increases intracellular reactive oxygen species levels, enhances more DNA damage after olaparib treatment, and induces a state of “BRCAness”, suggesting that TIGAR is a potential therapeutic target in ovarian cancer patients.

## Introduction

After the initial discovery that poly (ADP-ribose) polymerase (PARP) inhibitors cause synthetic lethality in cancer cells with BRCA1 or BRCA2 deficiencies^1,2^, a number of PARP inhibitors have now been approved by the Federal Drug Administration (FDA) as anti-cancer drugs for various cancers with either germline or somatic mutations in *BRCA1* and *BRCA2*. With more advances in the understanding of PARP inhibitors, it is now well accepted that PARP inhibitors are also active in cancers with the so-called “BRCAness” phenotype^3,4^, which are found in tumors with HRD resulted from alterations of components other than *BRCA1* or *BRCA2* in the HR pathway^5–9^. In addition, alterations in genes not directly related to the homologous recombination (HR) pathway, such as PTEN loss^10^ and translocation in TMPRSS2-ERG^11^ and EWSR1-FLI1^12^, also result in the increased sensitivity to PARP inhibitors, suggesting that other molecular pathways unrelated to the HR pathway may also contribute to PARP inhibitor sensitivity. Hence, a better understanding of genetic determinants that contribute to PARP inhibitor sensitivity will extend the clinical utility of PARP inhibitors.

One of the challenges in the further development of PARP inhibitors is to fully understand the molecular mechanisms contributing to PARP inhibitor sensitivity^13^. To address this challenge, several studies have reported candidate genes that cause synthetic lethality with PARP inhibitors from genome-wide RNAi profiling and functional studies^5,14,15^. These studies identified genes involved in DNA damage response (DDR) and repair pathways, such as BRCA1, NBN, FANCD, FANCC, RAD51, LIG3, RAD51C, RAD51D, RAD21, ESCO1, and SMC3, as well as genes involved in replication and cell cycle progression, such as MCM proteins, TOP3A, POLB, and CDK7 as modulators of sensitivity to PARP inhibitors^5^. However, the clinical benefit of targeting these genes to enhance sensitivity to PARP inhibitors is questionable given that chemical inhibition of these genes may likely sensitize normal cells as well as cancer cells to PARP inhibitors. Therefore, there is an urgent need to explore additional synthetic lethal targets for PARP inhibitors.

Clustered Regularly Interspaced Short Palindromic Repeats (CRISPR)-directed Cas9-mediated endonuclease activity can disrupt specific genetic sequences in the genome and provide a means to perform loss-of-function genetic screens^16–18^. When combined with sub-lethal doses of PARP inhibitor, this approach may allow identification of genetic factors that, when disrupted, contribute to PARP inhibitor sensitivity or resistance. Unlike RNAi approaches, CRISPR/Cas9 system provides more thorough depletion of target gene expression with less off-target effects when the guide RNA is appropriately designed^19–21^.

We therefore used CRISPR/Cas9 system to perform a genome-scale loss-of-function screen to identify modifiers of olaparib sensitivity in cancer cells. From this screen, we identified *C12ofr5*, a gene that encodes a metabolic regulator, *TP53*-induced glycolysis and apoptosis regulator (TIGAR), as a candidate that modifies olaparib sensitivity in cancer cells. We found that downregulation of TIGAR results in enhanced cytotoxic effects of olaparib. Our results indicate that TIGAR knockdown (KD) produces two complementary effects that enhance the sensitivity to olaparib. First, TIGAR KD inhibits pentose phosphate pathway (PPP) resulting in an increase of intracellular ROS that enhances DNA damage upon olaparib treatment. Secondly, TIGAR KD induces “BRCAness” by downregulation of BRCA1 and the  Fanconi anemia pathway. Finally, relevant to therapeutic effects, TIGAR KD inhibits cell growth by induction of cellular senescence. While the first two aspects provide mechanisms of how TIGAR downregulation sensitizes cancer cells to olaparib, the latter reveals a possibility of targeting TIGAR as a promising strategy to prevent cancer progression. Most importantly, these in vitro findings were further supported with data from The Cancer Genome Atlas (TCGA), which shows that TIGAR is amplified in different cancer types including ovarian cancer, and higher expression of TIGAR is associated with poor overall survival of patients with high-grade serous ovarian cancer. Collectively, these results indicate that TIGAR modifies cellular response to PARP inhibitors in cancer cells and presents a novel therapeutic target for developing cancer therapies.

## Results

### TIGAR identified as a modifier of olaparib sensitivity

**Fig. 1:**
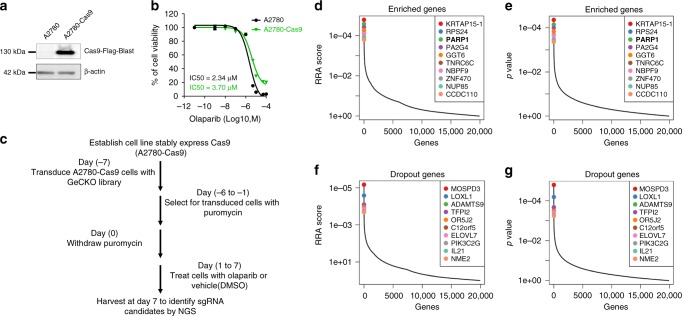
Genome-scale CRISPR knockout screening and candidate identification. **a** Stable expression of Cas9 endonuclease in A2780 cells. FLAG-tagged Cas9 was detected by western blot with anti-FLAG antibody. β-Actin was used as a loading control. **b** Olaparib sensitivity in parental A2780 and A2780-Cas9 cells. Dose-response curves were generated from SRB assays. IC50 was calculated in Prism 6 software. **c** The flowchart shows the time scheme of the CRISPR/Cas9 knockout screen. **d**–**g** Potential dropout and enriched candidates were identified after olaparib selection. The top 10 candidates that were most significantly enriched after olaparib selection are ranked by a modified robust ranking aggregation (RRA score) (**d**) or *p*-value (**e**). The top 10 candidates that were most significantly depleted after olaparib selection are ranked by RRA score (**f**) or *p*-value (**g**). Candidates were identified with the MAGeCK tool^23^ by comparing olaparib-treated groups with DMSO-treated groups

To identify modifiers of olaparib sensitivity, we first established a cell line that stably expresses Cas9 endonuclease (A2780-Cas9) by transducing A2780 cells with lenti-Cas9 viral particles followed by 2 weeks of blasticidin selection. Expression of Cas9 in this stable cell line is confirmed by western blot (Fig. 1a). The sulforhodamine B (SRB) assay shows that Cas9 expression and blasticidin selection has  a minimal effect on sensitivity to olaparib in A2780-Cas9 comparing to the parental A2780 cell (Fig. 1b). The screening was performed following the time scheme shown in Fig. 1c. We transduced the Cas9-expressing cells with Genome-scale CRISPR Knock-Out (GeCKO) Library B containing 58,031 guide RNAs (gRNAs) targeting 19,050 human genes with 3 gRNAs per gene. After 7 days of puromycin selection, cells expressing gRNAs were split into duplicates and treated with DMSO, 5 µM or 10 µM olaparib respectively. DMSO-treated cells served as a control. One week after DMSO or olaparib treatment, the  remaining cells were collected, gRNAs were amplified, and sequence library was prepared as previously described^16,22^. Amplicons were sequenced by Illumina next-generation sequencing, and by comparison of DMSO-treated and olaparib-treated groups, enriched or depleted gRNAs were identified using Model-based Analysis of Genome-wide CRISPR-Cas9 Knockout (MAGeCK) tool^23^. Top 10 candidates have been identified for enrichment (Fig. 1d, e) and depletion (Fig. 1f, g) analysis respectively. The enrichment analysis identifies candidate genes whose disruptions lead to olaparib resistance, meaning that these genes are potential candidates that increase sensitivity to olaparib. One of the candidate genes identified is *PARP1* (Fig. 1d, e). All three gRNAs show consistent, dose-dependent increase in abundance following olaparib treatment (Supplementary Fig. 1A). These results are consistent with a previous report indicating that PARP1 depletion results in resistance to PARP inhibitors^24,25^. While enrichment of gRNAs following olaparib selection indicates that target genes contribute to olaparib sensitivity, the depletion of gRNAs following olaparib selection indicates that target genes contribute to olaparib resistance. We identified *C12orf5* (*TIGAR*) as one of the genes that negatively modulates olaparib sensitivity (Fig. 1f, g), because gRNAs for *C12orf5* were depleted following olaparib treatment, suggesting that disruption of *C12orf5* gene sensitizes the cells to olaparib. We also identified *PI3K3C2G* and *NME2* as other candidate genes that decrease sensitivity to olaparib (Fig. 1f, g). Next, we transiently knocked down individual gene with pooled siRNAs to validate the potential candidates. We selected those candidates that have at least two different gRNAs being consistently and dose-dependently enriched or depleted after olaparib treatment for further validation, including *C12orf5* and *PARP1* (Supplementary Fig. 1A–B).

### TIGAR KD enhances olaparib sensitivity in cancer cells

**Fig. 2:**
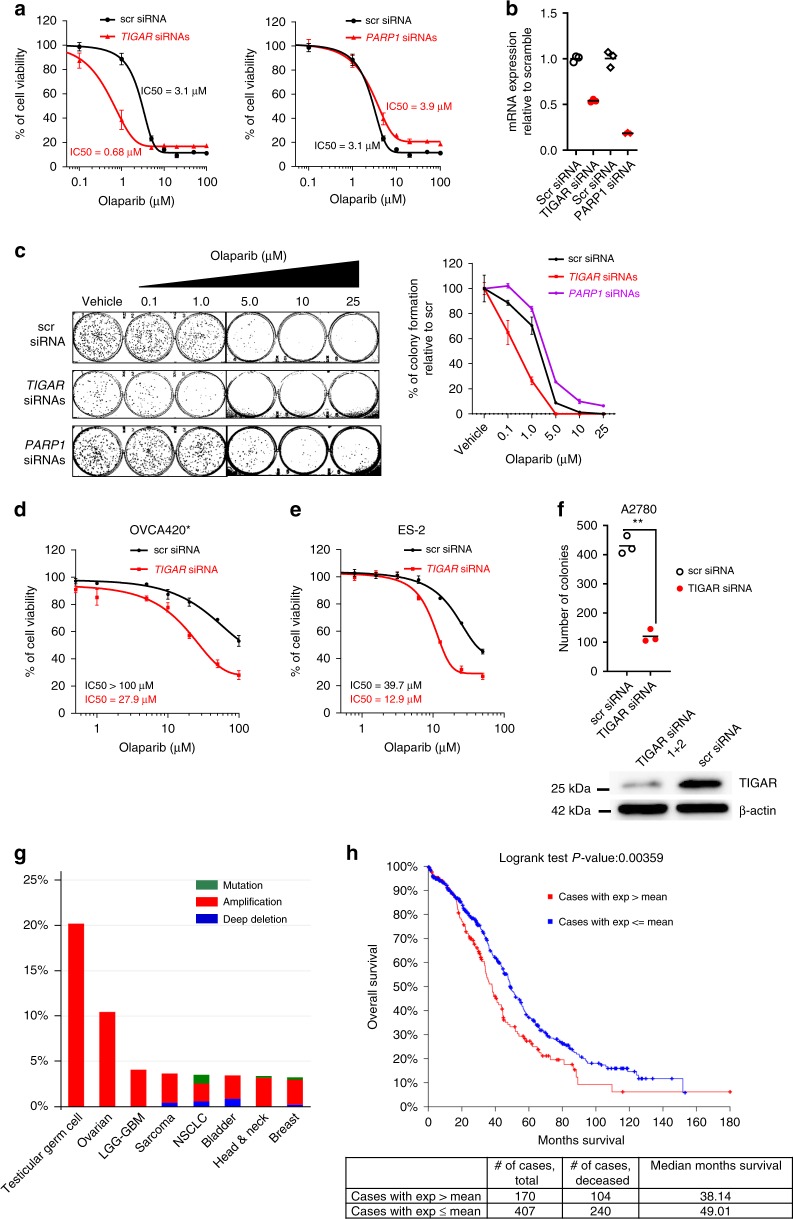
TIGAR knockdown with siRNAs sensitizes cancer cells to PARP inhibitor olaparib. **a** TIGAR knockdown (KD) resulted in a decrease while *PARP1* KD resulted in an increase in IC50 of olaparib in A2780 cells. The IC50 for olaparib was calculated from dose-response curves. **b** The real-time RT-PCR analysis demonstrates the KD of TIGAR and *PARP1* with corresponding pooled siRNAs. Three replicates are included. **c** Colony formation assay showed that TIGAR KD decreased the clonogenic survival of cells treated with olaparib and *PARP1* KD enhanced the clonogenic survival. Representative images of colonies are shown on the left. Quantification of colonies is shown on the right. **d**, **e** TIGAR siRNA KD increased sensitivity to olaparib in OVCA420* cell (**d**) and ES-2 cell (**e**). Data are shown as mean ± SEM. **f** TIGAR KD resulted in decreased colony formation. Three replicates are included. Statistical analysis was performed with Student’s *t*-test, **p < 0.01. **g** TIGAR was amplified in over 11% of ovarian tumor samples from TCGA dataset. (H) Higher TIGAR expression was associated with poor overall survival in high-grade serous ovarian cancer patients. A total of 577 patients from TCGA data set for high-grade serous ovarian cancer were included in the analysis. mRNA expression was quantified by RNA sequencing. TIGAR expression above the mean (Expression>Mean) was considered as overexpressed

To validate the selected candidates, we pooled two siRNAs for each gene and knocked down their expression in A2780 cell line that was used in the initial screen. Forty-eight hours after siRNA transfection, we performed SRB assay and colony formation assay to assess the sensitivity to olaparib (Fig. 2a–c). Results showed that a transient KD of TIGAR (Fig. 2b) leads to approximately fivefold decrease in IC_50_ of olaparib in A2780 cells (Fig. 2a). Clonogenic survival analysis also indicates a decrease in colony formation in TIGAR KD cells when combined with various concentrations of olaparib (Fig. 2c). Similar results were obtained with SRB assay in two additional cancer cell lines, which are less sensitive to olaparib (Fig. 2d, e), suggesting that downregulation of TIGAR sensitizes these cells to olaparib. Interestingly, we observed that TIGAR KD by itself significantly (**p < 0.01) decreased colony formation (Fig. 2f), and similar results were observed in another cancer cells OVCA420* (Supplementary Fig. 2A–B), suggesting that TIGAR depletion attenuates the growth of cancer cells. These observations are consistent with the previous report in glioblastoma cells^26^. These results suggest that TIGAR depletion attenuates the growth of cancer cells and enhances sensitivity to olaparib.

We were also able to validate the effect of *PARP1* KD on olaparib sensitivity. PARP1 KD showed a decrease in sensitivity to olaparib in A2780 cells (Fig. 2a–c). This is consistent with previous reports that depletion of PARP1 leads to PARP inhibitor resistance^24,25^. However, we were unable to validate the effect of other candidate genes on olaparib sensitivity (Supplementary Fig. 2C–D). It should be noted that we only observed a marginal decrease in sensitivity to olaparib with a transient KD of PARP1 by siRNAs, and this effect is not as dramatic as previous studies showing a 10-fold decrease in sensitivity to PARP inhibitor with the genetic disruption of PARP1 expression^24,25^. The difference may be the result of incomplete KD of PARP1 by siRNAs in our study. Differences in the efficiency of candidate gene suppression by siRNAs and CRISPR could be one reason explaining our failure to validate some candidate genes from the CRISPR knockout screen.

### TIGAR expression is associated with poor survival outcome

The analysis of datasets from TCGA indicates that TIGAR is amplified across different cancer types including ovarian cancer, in which it is amplified in over 11% of cases (Fig. 2g). TIGAR amplification is correlated with higher expression of TIGAR (Supplementary Fig. 2E). We also analyzed the RNA sequencing dataset of high-grade serous ovarian carcinomas from TCGA. We classified patients into two groups depending on the levels of TIGAR expression in the tumor: tumors with TIGAR expression above the mean are considered tumors with high expression of TIGAR whereas tumors with TIGAR expression at or below the mean are considered tumors with low expression of TIGAR. The analysis indicates that patients with high TIGAR expression in the tumor have a significantly (Logrank Test *p*-value = 0.00359)poor overall survival outcome compared to patients with low TIGAR expression in the tumor (Fig. 2h). Patients with high TIGAR expression have around 11 months lower median overall survival compared to patients with low TIGAR expression (*p* = 0.00359). Collectively, these results suggest an important role of TIGAR in cancer development and in modifying the response to PARP inhibitors. Finally, targeting TIGAR in cancer presents a potential strategy to overcome PARP inhibitor resistance and to prevent tumor progression.

### TIGAR KD enhances DNA damage and cytotoxicity of olaparib

**Fig. 3:**
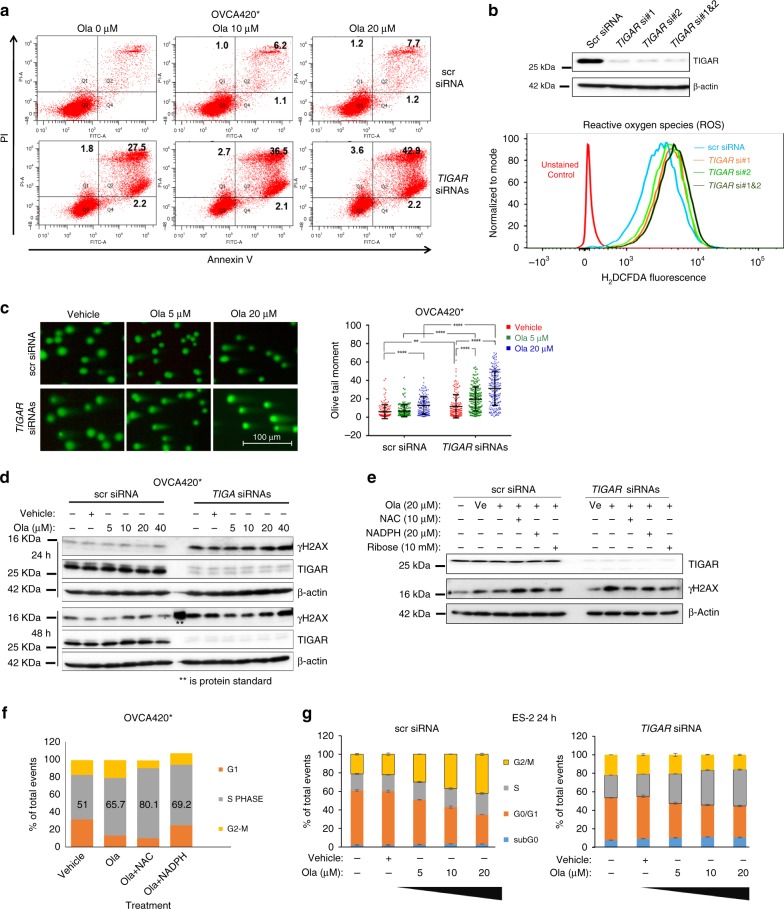
TIGAR KD induces apoptosis and enhances the cytotoxicity of olaparib through the increase of DNA damage. **a** Annexin V/PI flow cytometry profiles are from representative experiments with vehicle or olaparib (ola) treatment after control (scr) or *TIGAR* siRNA transfection. PI, propidium iodide. **b** Western blot indicates TIGAR KD with two different siRNAs and pooled siRNAs. β-Actin was used as a loading control. **c** DNA damage detected by neutral comet assay. A representative image for each group is shown on the top. Quantification data are shown in the graph on the right. Each dot represents one comet. Data are shown as median with SD. Statistics were conducted using one-way ANOVA analysis in Prism 6 software, **p < 0.01, ****p < 0.0001. **d** Western blot was performed with anti-γH2AX and anti-TIGAR antibodies at 24 and 48 h post-treatment with olaparib. β-Actin was used as a loading control. **e** Western blot showing γH2AX level following treatment with NAC, NADPH, or ribose after olaparib treatment in TIGAR KD cells. **f** Cell cycle analysis in parental OVCA420* cells following treatments shown on *X*-axis. **g** Cell cycle analysis in ES-2 cells 48 h after transfection and treatment with olaparib as indicated for an additional 24 h. Data are shown as mean ± SD

Next, we determined the mechanisms by which TIGAR downregulation leads to olaparib sensitivity. TIGAR shows sequence similarity to the bisphosphatase domain of Fructose-2,6-bisphosphatase^27^ and has been shown to negatively regulate glycolysis by lowering the intracellular levels of fructose-2,6-bisphosphate (F-2,6-BP), thereby promoting oxidative PPP shunt. PPP is critical for the production of NADPH and ribose-5-phosphate, which is a precursor for the synthesis of nucleotides^27,28^. TIGAR expression also protects cells from DNA-damaging reactive oxygen species (ROS) through regeneration of reduced glutathione via NADPH and provides some levels of protection from DNA damage-induced apoptosis^27–30^. Therefore, we tested the effect of TIGAR KD on apoptosis with or without olaparib treatment. With Annexin V/PI staining, we observed a dramatic increase of apoptotic cells after TIGAR KD with pooled siRNAs in two different cells (Fig. 3a, Supplementary Fig. 3A). The increase of apoptosis was further enhanced when combined with olaparib, suggesting that TIGAR KD can potentiate the cytotoxicity effects of olaparib via increase of apoptosis.

Given that TIGAR enhances antioxidant NADPH regeneration by promoting PPP, previous studies have shown TIGAR KD results in increased levels of intracellular ROS^27,29^. To confirm these results in our cell lines, we determined cellular ROS levels by H_2_DCFDA dye following the TIGAR KD. After the KD of TIGAR with individual siRNA or pooled siRNAs, we observed a higher H_2_DCFDA fluorescence compared to scrambled siRNA (scr siRNA)-transfected cells (Fig. 3b), indicating that downregulation of TIGAR increases ROS levels in the cells. Consistent with increased levels of ROS, neutral comet assay showed an impressive increase in DNA damage after TIGAR KD (Fig. 3c). Moreover, TIGAR KD significantly (***p < 0.001, ****p < 0.0001) enhanced DNA damage caused by olaparib treatments. Consistent with higher levels of DNA damage, cells with TIGAR KD displayed higher levels of γH2AX at 24 and 48 h following olaparib treatment than the cells transfected with scr siRNA (Fig. 3d). Yu et al. have shown that TIGAR KD increased DNA damage by inhibiting PPP in hepatocellular carcinoma cells^31^. To check if a similar mechanism is contributing to DNA damage following TIGAR KD, we supplemented cells with NADPH, ribose or N-acetyl cysteine (NAC) and observed a decrease in γH2AX levels by olaparib after TIGAR KD (Fig. 3e), suggesting that the enhanced DNA damage after TIGAR KD is at least partially due to a reduction of substrates from PPP. Paradoxically, we observed an increase in γH2AX levels in cells co-treated with NAC or NADPH and olaparib. Therefore, we performed cell cycle analysis of these cells following a co-treatment with NAC or NADPH and olaparib. In these cells, we observed an increase in the population of S-phase cells compared to DMSO or olaparib-treated cells (Fig. 3f). Thus, an increase in γH2AX in the co-treated cells may be due to an increase in the S-phase. We also performed cell cycle analysis in ES-2 cells with or without TIGAR siRNA KD in combination with olaparib treatment. In scr-siRNA-transfected ES-2 cells, olaparib treatment caused G2/M arrest due to G2 checkpoint activation caused by olaparib, and these results are consistent with a previous report^2^. In TIGAR KD cells, olaparib treatment caused the increase in accumulation of the S-phase cells (Fig. 3g). Similar results were observed at 48 and 72 h after olaparib treatment (Supplementary Fig. 3B–C). The S-phase delay instead of in G2/M arrest in TIGAR KD cells treated with olaparib is unique. These data are consistent with the notion that an increase in DNA damage after TIGAR KD requires more time to resolve the DNA damages during S-phase in TIGAR KD cells. In addition, TIGAR KD may also affect the expression of genes associated with S-phase progression, and the altered cell cycle-related gene expression may also contribute to the delay in S-phase progression.

### TIGAR KD decreases nicotinic acid and specific nucleobases

**Fig. 4:**
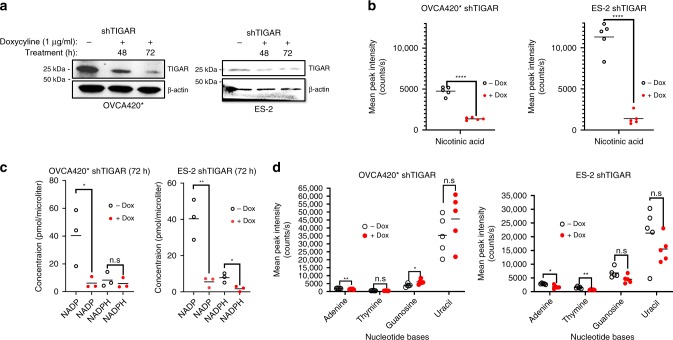
TIGAR KD decreases NAD+ precursor nicotinic acid, metabolites NADP and NADPH, and nucleobases. **a** Inducible TIGAR KD in OVCA420* and ES-2 at 72 h doxycycline treatment. **b** Levels of nicotinic acid obtained from the metabolomic profiling in cell lines following TIGAR KD. Five replicates are included. **c** The NADP+/NADPH assay results indicate a notable decrease of NADP in TIGAR KD cells compared to control TIGAR-proficient cells and an overall decrease in NADPH levels. Three replicates are included. **d** TIGAR KD also consistently decreases adenine biosynthesis in both cell lines and decreases other nucleobases in ES-2 cells. Five replicates are included. Data are shown as mean ± SEM. Statistics were performed with Student’s *t*-test, and *p* ≤ 0.05 was considered significant. *p ≤ 0.05, **p ≤ 0.01, ***p ≤ 0.001, ****p ≤ 0.0001

To assess the effect of TIGAR KD on cancer cell metabolism, we performed targeted metabolomic profiling of TIGAR KD cells stably expressing inducible shRNA against TIGAR. In two different cell lines OVCA420* and ES-2, doxycycline treatment efficiently downregulates TIGAR expression at 72 h (Fig. 4a). Targeted metabolomic profiling indicates nicotinic acid is consistently decreased in both cell lines following TIGAR KD (Fig. 4b). Consistent with these results, the NADP+/NADPH assay results indicate a significant (*p < 0.05, **p < 0.01) decrease of NADP+ and a consistent decrease of NADPH in TIGAR KD cells compared to control TIGAR-proficient cells (Fig. 4c). These results suggest TIGAR KD affects NAD+ biosynthesis pathway. TIGAR KD also consistently decreases adenine biosynthesis in both cell lines (Fig. 4d) and decreases other nucleobases in ES-2 cells (Fig. 4d). These results suggest TIGAR KD potentially affects nucleotide biosynthesis in these cells.

### TIGAR KD affects BRCA1 and Fanconi anemia pathway

**Fig. 5:**
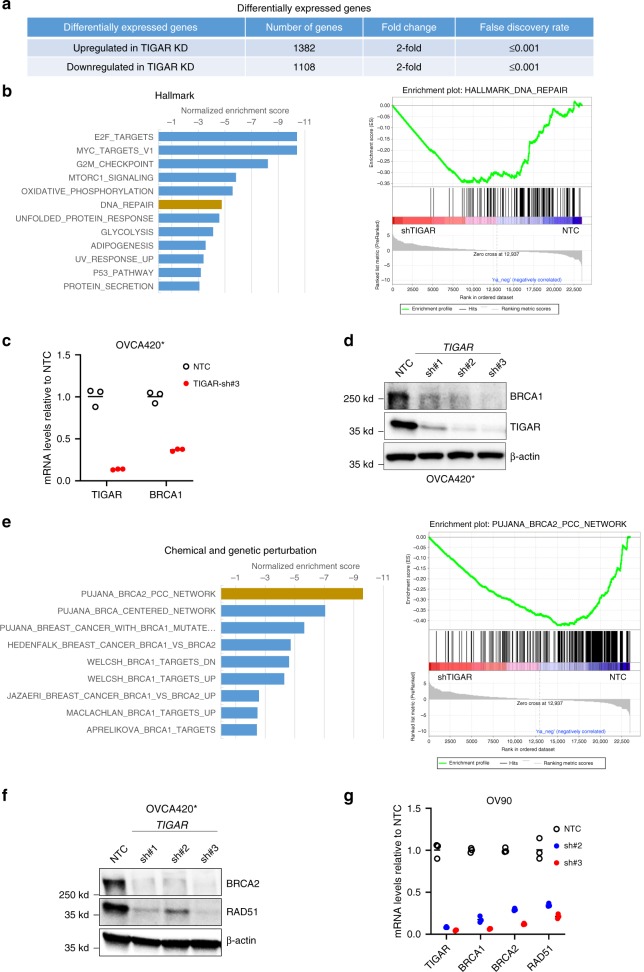
RNA sequencing analysis after TIGAR KD reveals downregulation of *BRCA1* and key DNA repair pathways. **a** Differentially expressed genes after TIGAR KD. 1382 genes were upregulated and 1108 genes were downregulated at least twofolds at a false discovery rate of ≤0.001. **b** GSEA analysis revealed a negative enrichment of the E2F targets gene set and DNA repair gene sets. **c**, **d** *BRCA1* was downregulated in TIGAR KD OVCA420* cells. qRT-PCR and Western blot were utilized to examine the expression of *BRCA1* mRNA and protein levels after TIGAR KD with shRNAs. For qRT-PCR, data shown as mean ± SEM. Results represent experiment with triplicates. **e** GSEA analysis also revealed negative enrichment of curated gene sets for DNA repair and BRCA1 and BRCA2 targets. **f**, **g** Fanconi anemia pathway components *BRCA2* and *RAD51* were downregulated after TIGAR KD with shRNAs. qRT-PCR was performed on OVCA420* and OV90 cells at 72 h after transduction of TIGAR shRNA. Data are shown as mean ± SEM. Results represent experiment with triplicates

Studies have shown that TIGAR modulates DDR after treatment with different DNA damaging agents^31^ and radiation^26^, and downregulation of TIGAR enhances DNA damages with these agents. Together with data presented above from our present study, these effects in large part depend on the reduction of PPP after TIGAR KD as reported previously^27,31^. To investigate whether there are other potential mechanism(s) involved in PARP inhibitor sensitivity after TIGAR KD, we performed RNA sequencing analysis in OVCA420* cells that were transduced with non-targeting control shRNA (NTC) or TIGAR-specific shRNA. The KD efficiencies of three different TIGAR shRNAs were variable (see Fig. 5d), and we used shRNA #3 that gave the most efficient KD. Following 54-hour transduction with lentiviral supernatant, we analyzed changes in the transcriptome using Illumina mRNA sequencing. A total of 1108 genes were downregulated by at least twofold at false discovery rate (FDR) ≤ 0.001 in TIGAR-KD cells compared to NTC-transduced cells. A total of 1382 genes were upregulated by at least twofold at FDR ≤ 0.001 in TIGAR-KD cells compared to NTC-transduced cells (Fig. 5a). The Gene Set Enrichment Analysis (GSEA) using the Hallmark gene sets indicates that the expression of E2F and MYC target genes as well as DNA repair genes were negatively affected by TIGAR KD (Fig. 5b). Interestingly, *BRCA1* was one of the noteworthy downregulated genes after TIGAR KD. Downregulation of *BRCA1* was validated with qRT-PCR and Western blot (Fig. 5c, d). The extent of *BRCA1* downregulation depends on the efficiency of TIGAR knock down, suggesting a dose-dependent effect (Fig. 5d). The GSEA with the curated gene sets for Chemical and genetic perturbations indicates the top transcription network negatively affected by TIGAR KD is the BRCA2_PCC Network (Fig. 5e). BRCA2_PCC network is defined by a set of genes that positively correlated with the expression of BRCA2. These results suggest that genes that are positively correlated with BRCA2 expression are downregulated upon TIGAR KD and that TIGAR KD also affect BRCA2 pathway. We also analyzed the differentially expressed genes using the Metascape bioinformatics web resources^32^. The annotation and enrichment analysis of genes downregulated by TIGAR KD indicates that the gene set is enriched for the Fanconi anemia pathway, DNA repair, DNA strand elongation, and cell cycle progression (Supplementary Fig. 4A). Consistent with both GSEA and the Metascape pathway analyses, we validated downregulation of genes involved in the Fanconi anemia pathway, such as *BRCA2* and *RAD51*, after TIGAR KD with two different shRNAs. BRCA2 and RAD51 expression were downregulated in TIGAR KD cells (Fig. 5f). A marked downregulation of BRCA1, BRCA2, and RAD51 was observed in another cell line infected with two different shRNAs (Fig. 5g). It is well accepted that downregulation of BRCA1 and BRCA2 leads to PARP inhibitor sensitivity, and our data suggest that TIGAR KD induces “BRCAness” by phenocopying the gene expression pattern produced by BRCA1/2 downregulation and thereby sensitizes cancer cells to olaparib. In general, the extent of downregulation of these genes is greater when TIGAR is more efficiently downregulated with shRNA. We also performed analysis for genes upregulated in TIGAR KD cells, and results indicate the enrichment of gene ontology for positive regulation of cell death, response to drug, regulation of PI3K activity, and positive regulation of intracellular signal transduction (Supplementary Fig. 4B). The upregulated genes involved in positive regulation of cell death is consistent with our observation of an increase of apoptosis after TIGAR KD (Fig. 3a and Supplementary Fig. 3A).

### TIGAR KD reduces DNA synthesis in cancer cells

**Fig. 6:**
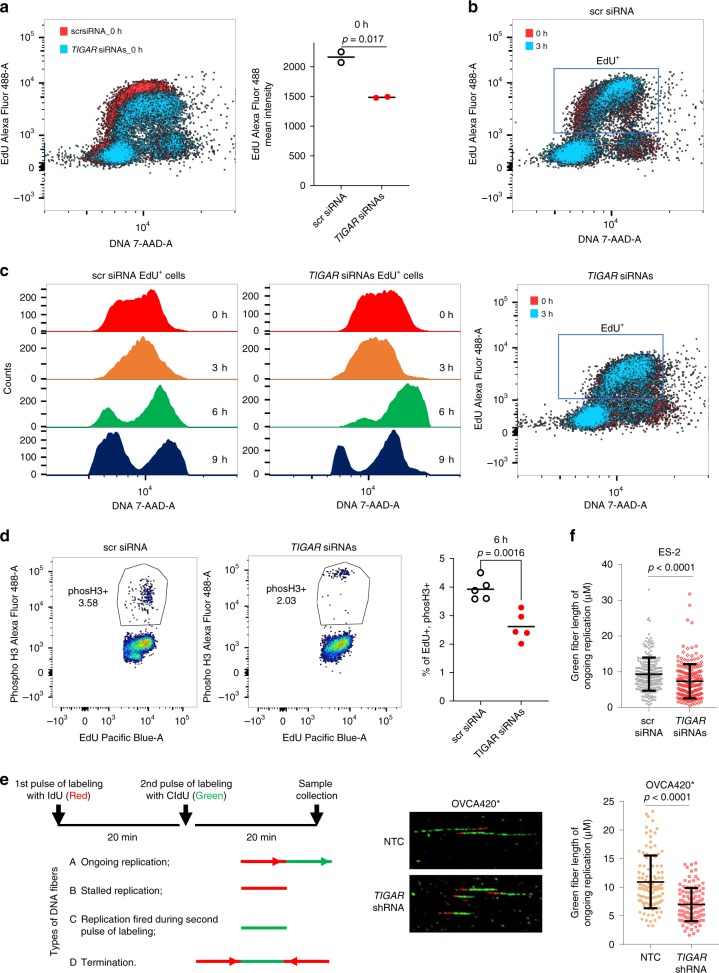
TIGAR KD leads to defects in DNA replication and cell cycle progression. **a**–**c** EdU labeling shows S-phase and cell cycle progression delay after TIGAR KD. **a** TIGAR KD results in less EdU incorporation. Representative plot is shown on the left, and the mean EdU intensity is shown on the right. Mean intensities were derived from two experiments consisting of at least 10,000 cells in each experiment immediately after EdU pulse labeling (0 h). **b** TIGAR KD leads to delay of S-phase progression. Representative plots were shown with comparison of 0 h and 3 h after EdU pulse labeling. **c** TIGAR KD delays cell cycle progression. Representative results were shown for EdU positive cells 0, 3, 6, 9 h after EdU pulse labeling. 7-AAD was used to detect DNA content. **d** EdU and phospho-Histone H3 double labeling reveals a decrease of mitotic cells in TIGAR KD cells. Samples were collected 6 h after EdU pulse labeling, fixed and stained to detect EdU and phospho-H3. On the left shows representative plots of cells with both EdU and phospho- H3 from scr or TIGAR siRNA treated groups, and right shows quantification results, five replicates are used. **e**, **f** DNA fiber combing assay indicates decreased of DNA replication with TIGAR KD. **e** Scheme of pulse labeling with IdU and CldU for fiber combing assay and four major types of DNA fibers. Representative images of DNA fibers from OVCA420* cells with NTC (Non-Targeting Control) shRNA or *TIGAR* shRNA were shown on the right. **f** Quantification of green fiber length from the ongoing replication in ES-2 cells with siRNA transfection (top) and OVCA420* cells with shRNA transduction (bottom). For ES-2 cells, around 200 fibers were quantified for each group, *N* = 2. For OVCA420*cells, 100 fibers were quantified for each group from one representative experiment. Quantification was done in Image J software (NIH). Statistics was done with student’s *t*-test, *p* < = 0.05 is considered as significant

Based on our RNA sequencing data analysis indicating that gene sets involved in DNA replication and cell cycle were notably downregulated in TIGAR KD cells (Supplementary Fig. 4A), we performed qRT-PCR analysis of candidate genes relevant for DNA replication and cell cycle progression in two cancer cell lines transduced with two different shRNAs. Results indicate that cyclin E2 (CCNE2), BLM, CDC45, MCM10, MKI67, and PCNA were downregulated in TIGAR KD cells (Supplementary Fig. 5A–B). These results suggest DNA synthesis might be negatively affected in TIGAR KD cells. To confirm this possibility, we performed EdU labeling of cancer cells following transient KD of TIGAR by siRNAs. The results indicate a remarkable decrease in EdU immunoflouresence in TIGAR KD cells compared to scrambled siRNA-transfected cells although EdU-positive (S-phase) cells are similar in both groups (Fig. 6a). Additionally, within 3 h of the pulse-chase, most of the cells in TIGAR-proficient, scrambled siRNA-transfected cells were progressed into G2/M phase whereas the majority of TIGAR KD cells remained in S-phase (Fig. 6b). Finally, at 6 h and 9 h of chase, substantially higher population of EdU-labeled cells reentered the G1 phase in scrambled siRNA-transfected cells compared to TIGAR KD cells (Fig. 6c and Supplementary Fig. 6A), indicating that there is a delay in cell cycle progression in TIGAR KD cells. In addition to delay in S-phase, G2/M transition is also affected in TIGAR KD cells because remarkably lower number of phospho-H3-positive M-phase cells were observed in TIGAR KD cells compared to TIGAR-proficient counterparts (Fig. 6d and Supplementary Fig. 6B). Finally, to demonstrate the effect on DNA synthesis, we performed DNA fiber assay using IdU and CldU. Quantitative analysis of the length of CldU-labeled DNA fibers indicative of ongoing replication (IdU incorporation followed by CldU incorporation, Fig. 6e) demonstrates that DNA synthesis in TIGAR KD cells is lower than TIGAR-proficient cells in two different cell lines (Fig. 6f). These results are consistent with the Metascape pathway analysis which indicated genes associated with DNA strand elongation is negatively affected by TIGAR KD (Supplementary Fig. 4A).

### TIGAR KD induces senescence and decreases clonogenicity

**Fig. 7:**
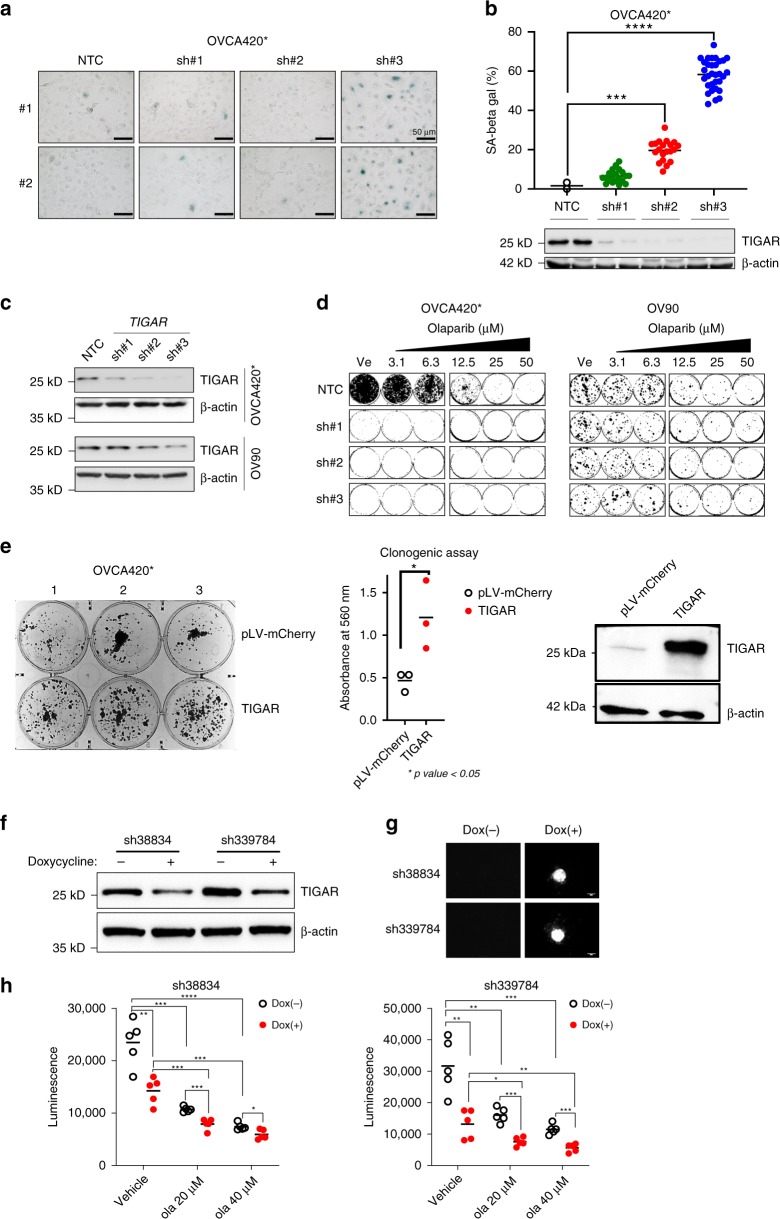
TIGAR KD increases senescence, decreases clonogenicity of cancer cells, and enhances sensitivity to olaparib. **a** TIGAR KD increases senescence in OVCA420* cells. SA-β-gal staining of OVCA420* cells transduced with NTC or TIGAR shRNAs. Representative images from one experiment are shown. **b** The dot-plot shows result from one representative experiment with duplicates. Images were taken from different areas in each well of the replicates and quantified for positive staining cells. Representative western blot shows TIGAR KD with individual shRNAs. **c** Western blot indicates efficient KD of TIGAR expression by shRNAs in OVCA420*. NTC, non-targeting control shRNA. β-Actin was used as a loading control. **d** Colony formation assay after TIGAR shRNA KD in OVCA420* and OV90 cells. 72 h after shRNA viral transduction, cells were seeded for clonogenic assay and treated with vehicle and increasing concentrations of olaparib. Representative images of colonies were shown. **e** Colony formation assay in OVCA420* cells with TIGAR expression rescue after TIGAR KD for 72 h in OVCA420* cells. Seventy-two hours after TIGAR siRNA KD, TIGAR and pLV-mCherry control were respectively expressed in the knocked down cells using viral transduction. Cells were seeded for clonogenic assay after 48 h of transduction and allowed to grow for 13 days. Representative images of SRB-stained colonies are shown, and the quantification of absolute SRB absorbance from triplicates is shown in a dot-plot. **f** Western blot analysis of TIGAR KD by two different shRNAs in OVCA420* after doxycycline (1 µg/mL) induction. **g** Representative images show induction of red fluorescent protein (RFP) with doxycycline within tumor spheroids and is used to monitor shRNA expression. **h** TIGAR KD enhances sensitivity to olaparib. Cell viability of spheroids was determined with CellTiter-Glo 3D reagents. Data are shown as mean with SEM. Statistics were performed with Student’s *t*-test, and *p* ≤ 0.05 was considered significant. *p ≤ 0.05, **p ≤ 0.01, ***p ≤ 0.001, ****p ≤ 0.0001

The previous report from Peña-Rico et al. showed that TIGAR KD with siRNA in glioblastoma cells induces senescence^26^. To confirm this effect in our ovarian cancer cell lines, we performed senescence-associated β-galactosidase (SA-β-gal) analysis in OVCA420* cells that were transduced with NTC shRNA or three different shRNAs targeting TIGAR. Consistently, we observed an increase in SA-β-gal staining in the TIGAR KD cells (Fig. 7a) and the extent of cells undergoing senescence is inversely correlated with TIGAR expression level (Fig. 7b). Similar results were observed in A2780 cells (Supplementary Fig. 7). These data suggest that more efficient KD of TIGAR leads to induction of senescence.

To check if an increase in senescence results in reduced clonogenicity, we used three different shRNAs to stably KD TIGAR expression in two different cancer cell lines, OVCA420* and OV90. Western blot analysis indicates three different shRNAs KD TIGAR expression with different efficiencies and were more effective in OVCA420* cells in general (Fig. 7c), which might be the result of different viral transduction efficiency between OVCA420* and OV90 cells. Colony formation assay indicates a marked decrease in clonogenicity following TIGAR KD in OVCA420* treated with DMSO (Fig. 7d). Similarly, a decrease in clonogenicity that corresponds with the levels of TIGAR KD was observed in OV90 cells (Fig. 7d). Clonogenic growth was further decreased by olaparib treatment in OV90 cells. These results suggest that efficient downregulation of TIGAR by itself may prevent cancer cells from growing, which indicates that TIGAR might be a good therapeutic target to explore.

### TIGAR KD enhances therapeutic effects of olaparib

Three-dimensional (3D) cell culture models are used as intermediate models between in vitro two-dimensional (2D) monolayer cancer cell culture and in vivo tumors^33^. Emerging evidence suggests that 3D culture systems represent more accurately the actual tumor microenvironment than 2D culture systems^34^, and it is considered to be a more representative model to test therapeutic effects of drugs in vitro^33–35^. We also validated the effect of TIGAR rescue post KD on clonogenic growth and found that cells with TIGAR re-expression post KD had an enhanced survival and growth in comparison to pLV-mcherry empty vector rescue (Fig. 7e). In order to test therapeutic effects of the combination of olaparib with TIGAR downregulation, we decided to use inducible shRNAs to perform the 3D spheroids formation assay. We first confirmed that induction of shRNA expression by doxycycline treatment was effective at knocking down TIGAR (Fig. 7f). Induction of shRNA can be monitored using RFP fluorescence (Fig. 7g and Supplementary Fig. 8A). Consistent with the results of reduced clonogenicity in 2D culture, we observed a significantly reduced (two-tailed Student’s *t*-test, *p < 0.05, **p < 0.01, ***p < 0.001, ****p < 0.0001) spheroid growth following TIGAR KD in 3D culture (Fig. 7h). With two different inducible shRNAs against TIGAR, we observed a notable decrease of cell viability in spheroids and sizes of spheroids when olaparib treatment was combined with TIGAR KD (Fig. 7h and Supplementary Fig. 8B). These data indicate that TIGAR KD enhances the therapeutic effects of olaparib, which is consistent with our findings in RNA sequencing analysis that BRCA1 and Fanconi anemia pathway were downregulated after TIGAR KD.

## Discussion

Several mechanisms have been proposed for PARP inhibitor resistance, such as the alterations that directly or indirectly restore HR function, including reversion mutation of *BRCA1/2*, epigenetic re-expression of BRCA1, and overexpression of 53BP1 and stabilization of partially truncated BRCA1^36–38^. Other non-HR mechanisms, such as loss of *PARP1* expression, PI3K/AKT pathway, and increase of drug transporters P-glycoprotein to facilitate efflux of drugs also contribute to PARP inhibitor resistance^24,25,39–41^. However, our knowledge about PARP inhibitor resistance is incomplete and more effective combination therapies need to be developed to overcome resistance. RNAi-based functional genetic screens are valuable techniques to efficiently identify a set of genes underlying a particular cellular process^42–45^. Although they are powerful tools to disrupt gene function, they suffer from low suppression of gene levels and frequent off-target effects, resulting in high rates of false positive and negative results^46–48^. CRISPR/Cas9-based system was shown to have advantages in these aspects, and pooled gRNA screens were effective in loss-of-function screens in mammalian cells as well as in vivo screens in mouse^16,18,49,50^. Several published studies reported siRNA or shRNA library screens to characterize modifiers of PARP inhibitor response^5,14^. Although our report is not the first to perform CRISPR/Cas9 screen to identify modifiers of PARPi response^51^, our study is the first to report TIGAR as a candidate modifier of the response to olaparib.

TIGAR was originally described as a p53-induced gene that regulates glycolysis and apoptosis^27^. However, subsequent studies described its additional role in DNA damage signaling, mitophagy, autophagy, and senescence^27,29,31^. In cancer cells, TIGAR plays an important role in regulating glycolytic flux and promotes NADPH and antioxidant regeneration through oxidative PPP^52^, and TIGAR KD results in a decrease in NADPH, an increase in ROS, and higher basal levels of DNA damage^27,31^. Previous report indicates that TIGAR KD results in senescence of glioblastoma cells^26^. Given its pivotal role in antioxidant regeneration, it is not surprising that our genome-scale CRISPR/Cas9 screen identifies *C12orf5* (TIGAR) as a dropout candidate gene involved in sensitizing cancer cells to PARP inhibitor olaparib.

Our screen indicates that all three specific gRNAs for TIGAR were dose-dependently depleted following exposure to 5 and 10 µM olaparib (Supplementary Fig. 1B). Our studies showed that TIGAR-deficient cells are more sensitive to olaparib (Fig. 2). TIGAR KD by RNAi resulted in increased levels of ROS and higher basal levels of DNA damage (Fig. 3b, c). Consistent with previous studies^2^, cancer cells show increased accumulation in G2/M phase when they are treated with olaparib. In contrast, TIGAR-deficient cells show increased accumulation in S-phase when they are treated with olaparib (Fig. 3f and Supplementary Fig. 3B–C). These results are consistent with our observation that TIGAR-deficient cells sustained more DNA damage, thereby requiring longer time to resolve DNA damages in S-phase. TIGAR KD also leads to growth inhibition supported by our observations of an increase in apoptosis (Fig. 3a and Supplementary Fig. 3A) and the induction of senescence (Supplementary Fig. 6). These results are consistent with the analysis of global changes in the transcriptome following TIGAR KD, which shows that genes involved in cell cycle progression were most notably affected by TIGAR KD (Fig. 5). The delayed S-phase progression and increased senescence in TIGAR KD cells may also be related to the effect on nucleobase biosynthesis because TIGAR KD affected adenine biosynthesis in two cell lines and biosynthesis of other nucleobase in ES-2 cell line.

Interestingly, TIGAR KD leads to downregulation of *BRCA1* (Fig. 5b, f). GSEA from RNA sequencing indicates TIGAR KD produces a gene signature that is similar to BRCA1 downregulated cancer cells (Fig. 5c). Moreover, pathway analysis showed that one of the significant GO terms associated with the downregulated genes is Fanconi anemia pathway (Fig. 5d). BRCA1 is a tumor suppressor that is involved in a handful of important cellular processes, among which its role in HR and transcriptional regulation in DDR draws the most attention^53,54^. BRCA1 deficiency leads to HR defects and sensitization of cancer cells to PARP inhibitor^2^. Fanconi anemia pathway regulates DNA repair by HR, and defects in Fanconi anemia pathway leads to DNA repair defects, resulting in low tolerance for DNA damaging agents^55^. Accordingly, downregulation of BRCA1 and Fanconi anemia pathway in TIGAR-deficient cells may provide a molecular mechanism attributable to the increased sensitivity to olaparib after TIGAR KD in cancer cells. The increased DNA damage and sensitivity to olaparib following TIGAR KD may also affect NAD+ biosynthesis because TIGAR KD decreases NAD+ precursor nicotinic acid in two cancer cell lines. NAD+ is an important metabolite for several pathways including redox pathway for the generation of NADP/NADPH redox pair as well as DNA repair pathway that utilizes poly ADP ribosylation.

It is important to note that due to pleiotropic role of TIGAR in cancer metabolism, TIGAR KD is expected to produce pleiotropic effects including the increase in DNA damage, likely contributed by the combined effects of deficiencies in ROS mitigation and DNA repair, and the increase in senescence, likely contributed by the combined effects of DNA damage and subsequent checkpoint activation, a decrease in nucleobase pool, and E2F transcription factor pathway inhibition. Collectively, multiple molecular mechanisms may contribute to senescence in TIGAR KD cells, and these mechanisms should be delineated in future studies.

Targeting TIGAR may induce “BRCAness” in cancer cells and serve as a combination strategy to overcome PARP inhibitor resistance or to enhance PARP inhibitor therapeutic effects. Our data from spheroids formation assay suggest that downregulation of TIGAR enhances therapeutic effects of olaparib (Fig. 6). In the future, it will be informative to test the effects of TIGAR downregulation in combination with PARP inhibitors in vivo. Importantly, TCGA data analysis indicates that higher expression of TIGAR is observed in many different cancer types, and patients with higher TIGAR expression show poor overall survival outcome in high-grade serous ovarian cancer (Fig. 7). This data suggests the clinical relevance of TIGAR expression in cancer, and targeting TIGAR might be a useful strategy to improve cancer treatments.

In addition to its effect on enhancing sensitivity to olaparib, an efficient KD of TIGAR in OVCA420* cells causes a decrease in DNA strand elongation, a decrease in DNA synthesis, accumulation of S-phase cells, and a decrease entry into G2/M-phase. Together with effects on DNA synthesis, TIGAR KD also causes an increase in cellular senescence and reduced clonogenicity. Collectively, these results highlight TIGAR as a potential therapeutic target in cancers.

Along with our present study, several studies have shown the potential of targeting TIGAR to enhance therapeutic effects of cancer therapies. These include direct targeting of TIGAR with siRNA^26^ or miR-144^56^, and indirect downregulation with a cell-penetrating peptide inhibitor of MUC1-C^57,58^ or c-Met TKIs^59^. Therefore, in the future, development of pharmacological methods to directly target TIGAR is an attractive direction to pursue. TIGAR is characterized as an F-2,6-BPase, which might be targeted with specific phosphatase inhibitors to inhibit its activity and inhibit PPP to enhance the cytotoxicity of cancer therapies as suggested with our data and reports from other groups^26,58^. Proteolysis targeting chimera (PROTAC)-based degraders that specifically targeting TIGAR can also be developed.

One potentially promising aspect of targeting TIGAR in cancer is that it regulates Warburg effect. A metabolic switch, known as Warburg effect, enables cancer cells to utilize higher amount of glucose by shunting glucose-6-phosphate to PPP and this PPP shunt is in part mediated by TIGAR. This metabolic feature is almost universal in cancer, and therefore may represent a critical step in cancer progression. The beneficial effect of this metabolic feature for cancer cells is that it provides critical substrates for DNA and thus enables replication as well as provides critical substrates for regeneration of antioxidants, thereby protecting cancer cells from excessive oxidative damages from accelerated cellular metabolism. Therefore, targeting TIGAR may not only deplete the cancer cells with critical substrates required for proliferation, but also deprive them with substrates required for regeneration of antioxidant. The combined effect of TIGAR inhibition result in increased ROS, DNA damage, cellular senescence, and increased sensitivity to PARP inhibitors.

## Methods

### Cell lines and cell culture

A2780 cells and ES-2 cells were maintained in MCDB105 and M199 (1:1) (Sigma, USA) containing 5% FBS (Sigma), OV90 cells were maintained in MCDB105 and M199 (1:1) with 15% FBS. OVCA420* cells were cultured in DMEM (Sigma and Caisson Labs, USA) supplemented with 10% FBS. All the media were supplemented with 100 units/MI penicillin and 100 µg/mL streptomycin. All cell lines were subjected to cell line identity confirmation. A2780-Cas9 stable cells were established by lentiviral transducing A2780 cells with pLenti-cas9 followed by selection with 400 µg/mL blasticidin for 2 weeks. ES-2, OVCA420* and OV90 cells were gifts from Dr. Viji Shridhar (Mayo Clinic). A2780 cell was from Dr. Andrew Godwin (The University of Kansas Medical Center). All experiments performed on cells that were passaged <20 times. Mycoplasma testing was performed during the studies, and cell cultures were free of mycoplasma. Cell line identification was performed at the end of experiments. OV90 showed 100% STR profiles matching to corresponding cell lines reported in ATCC or ExPASy. STR profiles for ES-2 and A2780 were performed in 2014 as a supplement to our recent publication^60^. STR profiles for OVCA420* does not match with any reported cell lines in ATCC, ExPASy, DSMZ, or CLIMA, and therefore we placed an asterisk to differentiate it from the original cell line.

### Pooled genome-wide CRISPR screen

Genome-scale CRISPR knock-out (GeCKO) v2.0 pooled libraries (Two vector system) were purchased from Addgene. GeCKO library B was amplified and prepared as previously described^16,22^. A2780 cells were infected with lentiviral particles of lenti-Cas9 at a multiplicity of infection (MOI) of ~1.0 followed by selection with blasticidin for 2 weeks to obtain the stable cell line expressing Cas9. The stable cells were then infected with the amplified lentiviral library B at the MOI of ~0.3. Thirty-million cells were used and seeded in 100 mm dishes with 3 × 10^6^ cells per dish. Puromycin was added the second after lentiviral library infection and kept for 7 days. After selection, surviving cells were pooled together and split into three groups with duplications to treat with DMSO, 5 or 10-μM olaparib for 1 week. For each treatment, 3×10^7^ cells were used per replicate. Genomic DNA was extracted and PCR was performed to prepare sequencing library as described before^16^. The library was sequenced using a HiSeq 2500 (Illumina). Data analysis was performed by MAGeCK tool^23^.

### Lentiviral particle production

Viral particles were produced by transient transfection of specific plasmids (GeCKO library B, lentiCas9-Blast, individual shRNA expressing plasmids) with psPAX2 and pMD2.G (Addgene) into HEK293T cells using Lipofectamine 2000 (Life Technologies). Media was collected 48 h after transfection and centrifuged at 2000 rpm at 4 °C for 10 min to pellet cell debris. The supernatant was filtered through a 0.45 μm low protein binding membrane (Millipore Steriflip HV/PVDF). Aliquots were stored at −80 °C.

### Antibodies and compounds

Mouse monoclonal anti-FLAG HRP conjugated antibody (A8592) was purchased from Sigma. Rabbit polyclonal anti-TIGAR antibody (GTX110514) was from GeneTex (Irvine, CA, USA). Rabbit anti-Phospho-Histone H2A.X (Ser139) antibody (#2577) was purchased from Cell Signaling Technologies (Danvers, MA, USA). Rabbit polyclonal anti-BRCA1 antibody (C-20, sc-642) was purchased from Santa Cruz Biotechnology (Santa Cruz, CA, USA). Mouse monoclonal anti-beta-actin antibody (A1978) was from Sigma-Aldrich (St Louis, MO, USA). For Secondary antibodies, horse anti-mouse IgG-HRP antibody (7076S) was purchased from Cell Signaling Technologies, Goat anti-rabbit IgG-HRP antibody (sc-2030) was from Santa Cruz Biotechnology. Olaparib (AZD2281, Ku-0059436) was purchased from Selleckchem. Olaparib stock solutions were made with DMSO at 50 mM and stored at −80 °C.

### Immunoblotting

Cells were washed at least twice with PBS at the end of treatments if applicable and then lysed with an appropriate volume of 1× electrophoresis sample buffer (Bio-Rad Laboratories, CA, USA) with 5% β-mercaptoethanol (Sigma-Aldrich). The cell lysates were then boiled at 95 °C for 5 min before using. Immunoblotting procedures were performed as previously described^60^. Equal amounts of total proteins were loaded for SDS-PAGE and transferred onto PVDF membranes (GE healthcare). All uncropped blots are available in Supplementary Fig. 9.

For the experiments shown in Fig. 3e, 48 h after transfection cells were pretreated with 10 µM NAC, 20 µM NADPH or 10 mM ribose respectively for 2 h, followed by 24 h treatment with vehicle or 20 µM olaparib.

### siRNA transfection

Gene-specific siRNAs and Scrambled negative control siRNAs were synthesized by Integrated DNA Technologies (IDT, Coralville, IA, USA). 3.5 × 10^5^ cells/well were seeded in 6-well plates and incubated at 37 °C overnight. Next day, 20 nM of each siRNA was transfected into the cells with Oligofectamine Transfection Reagent (12252011, Invitrogen) according to manufacturer’s instructions. Culture media was added 6–8 h after transfection without washing cells. Twenty-four to forty-eighthours after transfection, the transfected cells were trypsinized and seeded on 96-well plates (for cytotoxicity assay) or 6-well plates (for colony formation assay). Drugs were added around 12 h after seeding. For cytotoxicity assay, cells were incubated with drug for 3 days before measurement of cell viability using SRB assay. For colony formation assay, cells were exposed to drugs for 3 days and then changed to fresh media without drug until colonies formed and stained with SRB for imaging. To check the downregulation of gene expression, the transfected cells were collected to extract total RNA for qRT-PCR or proteins for western blot analysis 72 h after transfection. The sequences of siRNAs used are shown in the Supplementary Table 1.

### qRT-PCR

The total RNA was extracted with the Trizol reagent (15596–028, Invitrogen) according to the manufacturer’s manual. The cDNA was synthesized using SuperScript II reverse transcriptase (180604014, Invitrogen) with 1 μg of total RNA in a 20 μL reaction. The resulting cDNA was diluted 1:20 in nuclease-free water and 1 μL was used per qPCR reaction with triplicates. qPCR was carried out using Power SYBR Green PCR Master Mix (4367659, Thermo Fisher Scientific) on a CFX384 Real-Time PCR Detection System (Bio-Rad) including a non-template negative control. Amplification of GAPDH or 18S rR\NA was used to normalize the level of mRNA expression. Primers used in the assays are shown in the Supplementary Table 2.

### shRNA transduction

shRNAs for TIGAR were purchased from Sigma (SHCLNG-NM_020375). Lentiviral particles for shRNAs were produced as described above. Viral supernatant collected at 48 hours was used to transduce OVCA420* or OV90 cells. Experiments were conducted 2 days after viral particle transduction.

### Cytotoxicity assay using SRB

SRB assays were performed as previously described^61,62^, with modifications shown below. Three thousand cells/well were seeded in 96-well plates and treated with drugs at least 12 h after seeding. Then the cells were incubated for another 3 days. Dose-response curves were fitted and the IC50 for each drug was determined using GraphPad Prism 6 four parameters. All curves were constrained with 100% on top.

### Colony formation assay

A total of 500–1000 cells were seeded in 6-well plates and were treated with drugs at least 12 h after seeding and further incubated for another 3 days before changing to fresh media. The medium was changed every 2–3 days to allow colonies to form. At the end of experiments, SRB assay was performed to stain the colonies which were imaged with Molecular Imager Gel Doc XR System (Bio-Rad). The colonies were further dissolved and measured with a plate reader. The analysis of colonies was performed in GraphPad Prism 6. To perform the rescue experiments, cells were transduced with the control pLV-mCherry viral particles or pReceiver-Lv216 (Genecopoeia) viral particles containing the TIGAR open-reading frame and mCherry-IRES2-puromycin. Seventy-two hours later, cells were transfected with TIGAR siRNA targeting the 3′ untranslated region (3′ UTR) and subjected to colony formation assay.

### Apoptosis analysis with Annexin V/PI staining

Cells were transfected with scrambled siRNA or TIGAR siRNA and reseeded into 6-well plates around 24 h after transfection, followed by treatment with Vehicle, 10 μM or 20 μM of olaparib for additional 48 h. Cells were then subject to Annexin V/PI staining following manufacturer’s instruction (#640906, Biolegend). Briefly, cells were washed twice with PBS and stained with Annexin V and propidium iodide solution for 15 min at room temperature in the dark before analysis by flow cytometry.

### Measurement of intracellular ROS

Intracellular ROS was measured by using the oxidant-sensitive fluorescent probe 5-(and-6)-chloromethyl-2′,7′-dichlorodihydrofluorescein diacetate acetyl ester (CM-H2DCFDA, Molecular Probes). Forty-eight hours after transfection with scramble siRNA or *C12orf5* siRNA, cells were trypsinized, washed twice with PBS and incubated with 5 μM or CM-H2DCFDA in PBS at 37 °C for 30 min prior to flow cytometry analysis. Propidium iodide was added 10 min before flow analysis to exclude dead cells from ROS analysis.

### Neutral comet assay

Neutral comet assay was performed according to Tregvigen Instructions for Comet Assay with modifications. Briefly, 48 h after siRNA transfection, cells were treated with vehicle, 5 μM or 20 μM olaparib for 24 h. Cell suspension in PBS with 1 × 10^5^ cells/mL was mixed with molten LMA agarose (Trevigen) at a ratio of 1:10 and transferred onto Comet Slide (Trevigen). After cooling slides at 4 °C for 10 min, slides were immersed in Lysis solution (Trevigen, 4250–050–01) at 4 °C for 1 h and immersed in 50 mL of 1× Neutral Electrophoresis Buffer (0.5 mM Tris Base and 1.5 mM Sodium Acetate) for 30 min at 4 °C. 1 h electrophoresis was performed at 4 °C using Wide Mini-Sub Cell GT Horizontal Electrophoresis System (Bio-Rad) and applied voltage at 17 volts. Slides were then immersed in DNA Precipitation Solution (1M NH4Ac and 82.27% ethanol) for 30 min at room temperature followed by 30 min incubation in 70% ethanol. Then let slides dry at 37 °C for 10–15 min and stained with SYBR Green I and viewed slides and imaged comets using ZEISS fluorescent microscope (10×). Comets were analyzed using free comet assay software from Casp Lab (1.2.3beta2 version). Olive Tail Moment = (Tail.mean − Head.mean) × Tail%DNA/100.

### Cell cycle analysis

Cells were transfected with scr siRNA or TIGAR siRNA and reseeded into 6-well plates around 24 h after transfection, followed by treatment with different concentrations of olaparib for additional 24, 48 or 72 h. After treatment, cells were washed twice with 1× PBS and trypsinized before centrifuging at 1000 rpm at 4 °C for 10 min in 15 mL conical tubes. Then resuspend in 300 µL of ice-cold PBS after wash and fixed cells by adding 300 µL of ice-cold 95% ethanol dropwise and keep in dark at 4 °C to fix overnight. The next day cells were rehydrated, washed twice with ice-cold PBS and incubated in RNase A solution (1 mg/mL) at 37 °C for 15 min. Cells were then stained with 50 μg/mL propidium iodide solution in dark at room temperature for 15 min before flow cytometry analysis.

### DNA replication analysis with EdU labeling

For EdU labeling assay, 48 h after siRNA transfection, cells were seeded into 6-well plates, and pulse labeled with 10 µM of EdU for 1 h followed by sample collection at different time points, ea. 0, 3, 6, 9 h after washing off of EdU. EdU were detected with Click-iT EdU Flow Cytometty Assay kits with Alexa Fluor 488 picolyl azide (ThermoFisher Scientific, C10425) or Pacific Blue picolyl azide (ThermoFisher Scientific, C10636). Briefly, cells were washed in 1% BSA/PBS and fixed with Click-iT fixative for 15 min, followed by 15 min permeabilization with saponin-based permeabilization and wash buffer before Click-iT reaction to detect EdU, then cells were resuspended in permeabilization buffer and 0.25 µg/mL of 7-AAD (Invitrogen, catalog # 00–6993) were added 5–10 min before flow cytometry analysis on BD LSRFortessa. For detection of M-phase cells with mitotic marker, phospho-Histone H3, Alexa Fluor 488 conjugated rabbit anti-phospho-Histone H3 (Ser10) monoclonal antibody (Cell Signaling Technology, 3465S) was used.

### DNA fiber experiments

Fiber combing assay was performed as previously described^63^. Briefly, cells were seeded 48–72 h after siRNA transfection or shRNA transduction the day before replication labeling, then cells were pulse labeled with 100 µM IdU (Sigma-Aldrich, 17125) for 20 min, washed with PBS, followed by a 20 min 100 µM CldU (Sigma-Aldrich, C6891) pulse. Cells were trypsinized, pelleted and resuspended in low-temperature melting agarose for plugs formation. DNA was prepared with FiberPrep DNA Extraction Kit (GenomicVision, EXTR-001) followed by DNA fibers were combed on silanized coverslips (GenomicVision, COV-002) using the Genomic Vision combing machine following manufacturer’s instructions. Staining of DNA fibers was performed as follows: coverslips were dehydrated in an oven at 60°C for 2 h followed by sequential dehydration in 70%, 90% and 100% ethanol and denatured in a 0.5 M NaOH/1 M NaCl solution. After washing three times with PBS, coverslips were incubated at 37 °C for 30 min with 5% BSA in PBS, 1.5 h with primary antibodies: mouse anti-BrdU (1:25, BD Biosciences, 347580); rat anti-BrdU (1:100, Abcam, ab6326), and 1 h with secondary antibodies: sheep anti-mouse IgG Cy3 (1:500, Sigma-Aldrich, C2181); goat anti-rat IgG Alexa 488 (1:400, ThermoFisher Scientific, A-11006). Stained DNA fibers were mounted in Vectashield (Invitrogen), and images were obtained using a Zeiss AxioObserver LSM 800 with Airyscan microscope with a ×40 oil immersion objective lens. IdU was set to be red and CldU to be green. Fiber length was measured in Image J software (NIH). Supplementary Fig. 10 contains additional images.

### Senescence-associated β-galactosidase assay (SA-β-Gal)

Two days after transducing with individual TIGAR shRNAs, cells were trypsinized and reseeded in 6-well plates at the plating density of 1 × 10^5^ cells per well and incubated at 37 for 24 h. SA-β-Gal staining was performed as described before for adherent cultured cells (Itahana, Campisi et al. 2007). Briefly, cells were washed twice with 1× PBS, fixed with freshly prepared 3.7% formaldehyde in PBS for 5 min at room temperature, and washed twice with PBS. Cells were incubated in 2 mL of X-gal staining solution [1 mg/mL of X-gal, 40 mM citric acid/sodium phosphate buffer (pH 6.0), 5 mM potassium ferricyanide (Sigma, St. Louis, MO), 5 mM potassium ferrocyanide (Sigma), 150 mM NaCl, and 2 mM MgCl_2_] at 37 °C for 16 h before taking images with 10× objective under Leica DMI3000 B Inverted Phase Contrast Microscope. SA-β-Gal stain-positive cells were quantified in Image J software (NIH).

### Spheroid formation assay

OVCA420* cells stably expressing inducible TIGAR shRNAs (TRIPZ inducible lentiviral shRNAs were purchased from Dharmacon, US) were established by selection with puromycin after viral transduction. Stable cells were treated with 1 µg/mL doxycycline for 72 h to induce expression of shRNAs. Then 3000 cells/well were seeded into 96-well plates (plates were pre-coated with 100 µL 1.5% (wt/vol) agarose) and treated with vehicle or different concentrations of olaparib for 10 days. At the end of the experiment, pictures of spheroid were taken under a microscope and cell viability was measured with CellTiter-Glo 3D reagent (Promega Corporation).

### Targeted metabolomic profiling and analysis

Primary metabolites were analyzed by gas chromatography-time of flight mass spectrometry (GC-TOF MS). Five biological replicates from ES-2 TIGAR shRNA (-Dox), ES-2 TIGAR shRNA (+Dox), OVCA420* TIGAR shRNA (-Dox) and OVCA420* shRNA (+Dox) were submitted respectively (10 million cells per sample). Samples were homogenized using a GenoGrinder2010 (SPEXSamplePrep, Metuchen, NJ, USA) for 30 s at 1500 rpm and centrifuged at 14,000 × *g* for 2 min Cells were not washed before the homogenization step to avoid metabolic perturbation during the wash step. Cells were extracted with 1 mL of −20 °C cold, degassed acetonitrile:isopropanol:water (3:3:2, v/v/v). Supernatant (500 µL) was evaporated to dryness using a CentriVap (Labconco, Kansas, MO, USA). Metabolites were derivatized in two steps: first, carbonyl groups were protected by methoximation; second, acidic protons were exchanged against trimethylsilyl-groups to increase volatility. A 0.5 µL sample was injected with 25 s splitless time on an Agilent 6890 GC (Agilent Technologies, Santa Clara, CA, USA) using a Restek Rtx-5Sil MS column (30 m × 0.25 mm × 0.25 µm) with 10 m guard column (10 m × 0.25 mm × 0.25 µm) and 1 mL/min helium gas flow. Oven temperature was held at 50 °C for 1 min and then ramped up to 330 °C at 20 °C/min and held for 5 min Data were acquired at −70 eV electron ionization at 17 spectra/s from 85 to 500 Da at 1850 V detector voltage on a Leco Pegasus IV time-of-flight mass spectrometer (Leco Corporation, St. Joseph, MI, USA). The transfer line temperature was held at 280 °C with an ion source temperature set at 250 °C. Standard metabolite mixtures and blank samples were injected at the beginning of the run and every ten samples throughout the run for quality control. Raw data were preprocessed by ChromaTOF version 4.50 for baseline subtraction, deconvolution, and peak detection. Specifically, 3 s peak width, baseline subtraction just above the noise level, automatic mass spectral deconvolution, and peak detection at signal/noise levels of 5:1 throughout the chromatogram were used. Binbase version 5.0.3 was used for metabolite annotation and reporting. The following settings were used by the Binbase algorithm (rt × 5): validity of chromatogram 107 counts/s, unbiased retention index marker detection, MS similarity >800, retention index calculation by 5th order polynomial regression, retention index window 2000 units, and validation of unique ions and apex masses. Raw Metabolomic profiling data can be found at https://osf.io/d7pmc/ and in Metabolights as https://www.ebi.ac.uk/metabolights/MTBLS1113.

Analysis and calculations: The normalized Mean Peak Intensity values of the different Metabolites were compared using MetaboAnalyst 4.0 and *p* values were calculated using two-tailed student *t* test with unequal variance. Differentially expressed Metabolites were further analyzed using Graphpad Prism version 8. Results were expressed as the mean ± standard error of the mean (SEM) and differences between the two cell lines with and without doxycycline mediated TIGAR KD were analyzed by two-tailed Student’s *t*-test. *p* < 0.05 was considered to be statistically significant. *p < 0.05, **p < 0.01, ***p < 0.001, ****p < 0.0001.

### NADP/NADPH quantification assay

Cells were treated with and without doxycycline in three biological replicates and three technical replicates for 72 h before experiments. 1 × 10^6^ cells were harvested from each sample and cells were extracted using the NADP/NADPH extraction buffer by following the manufacturer’s instructions of NADP/NADPH Quantification Kit (MAK038, Sigma-Aldrich). Cell lysates were deproteinized by filtering through 10kDa cut-off spin filter (Amicon® Ultra 10K device). NADP and NADPH were independently quantified as per the manufacturers protocol and readings were obtained colorimetrically at an absorbance of 450 nm. Calculations and normalization of raw values was performed as per the manufacturer’s instructions (MAK038, Sigma-Aldrich). Normalized concentration values were then analyzed in GraphPad prism version 8 and differential levels of NADP and NADPH were expressed as the mean ± standard error of the mean (SEM). Statistical significance was calculated using two-tailed Student’s *t*-test. *p* < 0.05 was considered to be statistically significant. *p < 0.05, **p < 0.01, ***p < 0.001, ****p < 0.0001.

### RNA-sequencing analysis

Total RNA was extracted 54 h after cells being transduced with TIGAR shRNA viral supernatant. One microgram of total RNA was used to synthesize cDNA and sequencing library was prepared using Illumina TruSeq RNA Sample Preparation Kit v2 following manufacturer’s instructions. Cells transduced with non-targeting shRNA serves as a control. RNA-seq libraries were generated from three replicates from the control and experimental groups. Next-generation sequencing was performed with NextSeq 500 by Oklahoma Medical Research Foundation (OMRF) Clinical Genomics Center (OK, USA). FASTQ sequences were mapped to the human reference genome (hg19) using the CLC Genomics Workbench (v.10.0.1). Differentially expressed genes in TIGAR KD cells were determined by CLC Genomics Workbench using the Differential Expression for RNA-seq toolbox. This toolbox use Generalized Linear Model, and read counts are fitted to a negative binomial distribution. Differentially expressed genes were further analyzed with Metascape to identify biological pathways identified by differentially expressed genes^32^. In addition, logranked gene expression between TIGAR KD and non-targeting shRNA-transduced cells were analyzed using the Gene Set Enrichment Analysis to identify enriched pathways and gene sets using H: Hallmark gene sets and C2.CGP gene sets from MSigDB collections^64,65^.

### Statistics and reproducibility

All experiments were conducted in three technical replicates and three biological replicates respectively unless indicated otherwise. All data were analyzed using GraphPad Prism (ver. 6) unless indicated otherwise. Results from three biological replicates were expressed as the mean ± standard error of the mean (SEM) or standard deviation of the mean (SD). Differences between treatment regimens were analyzed by one-way ANOVA or two-tailed Student’s *t*-test. *p* < 0.05 was considered to be statistically significant. *p < 0.05, **p < 0.01, ***p < 0.001, ****p < 0.0001.

### Reporting summary

Further information on research design is available in the Nature Research Reporting Summary linked to this article.

## Supplementary information

Supplementary information

Reporting Summary

## Data Availability

Data that support the findings of this study are available at the following sites. Raw fastq files can be accessed via EBI Array Express at the following link. https://www.ebi.ac.uk/arrayexpress/experiments/E-MTAB-7284/?query=chien Pathway analysis data and any additional data used for generating figures can be accessed via OSF.io https://osf.io/d7pmc/files/ Raw Metabolomic profiling data can be found at https://osf.io/d7pmc/ and in Metabolights at https://www.ebi.ac.uk/metabolights/MTBLS1113.
