# Supplementary material for: Genome-scale CRISPR knockout screen identifies TIGAR as a modifier of PARP inhibitor sensitivity
Source: Commun Biol. 2019 Sep 9;2:335. doi: 10.1038/s42003-019-0580-6 (PMC6733792; doi:10.1038/s42003-019-0580-6)
Supplement: Supplementary file 1 — Supplementary information [file 42003_2019_580_MOESM1_ESM.pdf]

Supplementary Figure 1.

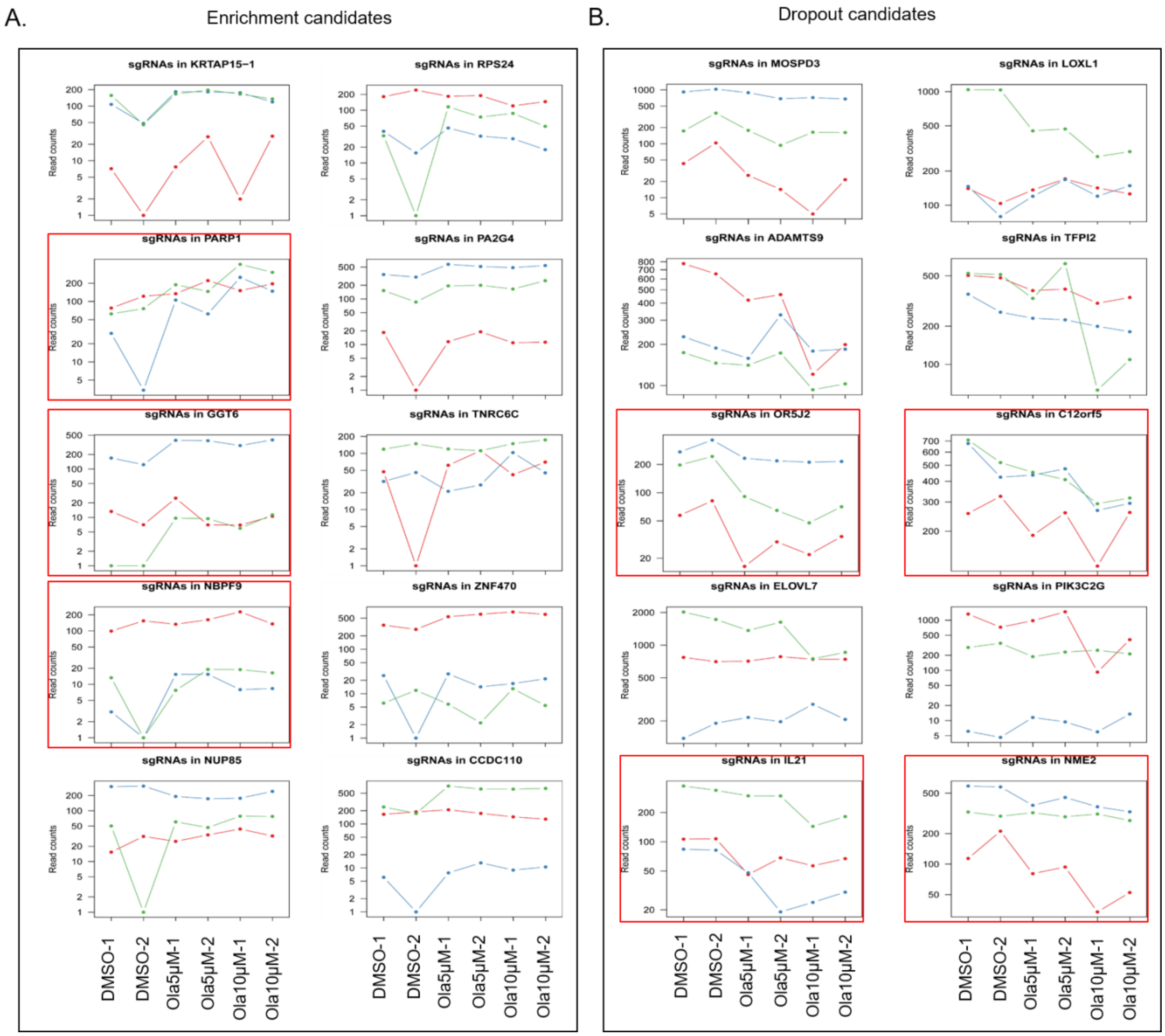

Supplementary Figure 1. Identification of potential candidates. (A) Profiles of enrichment candidates. Three different gRNAs for each candidate gene are shown in red, green and blue. Each data point represents corresponding treatment condition: DMSO-1, DMSO-2, olaparib 5 μM-1, olaparib 5 μM-2, olaparib 10 μM-1 and olaparib 10 μM-2, suffixed with replicate ID. For dropout candidates, gRNA read count decreases with olaparib treatment in a dose-dependent manner, such as C12orf5. **(B)** Profiles of dropout candidates. For the enriched candidate, gRNA read count increases with olaparib treatment in a dose-dependent manner, such as PARP1. Candidates with red boxes were selected for further validation with pooled siRNAs

Supplementary Figure 2.

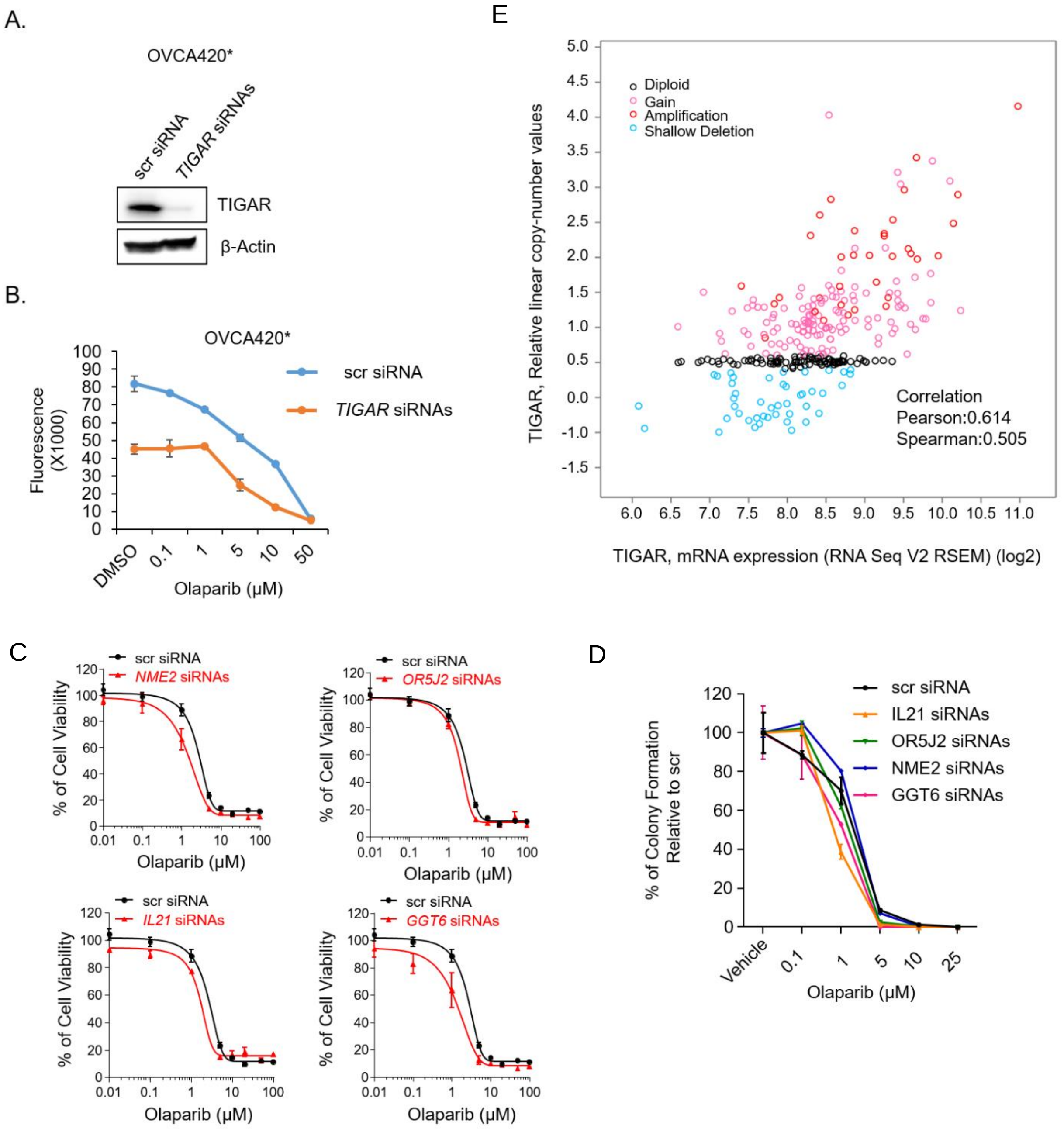

Supplementary Figure 2. Validation of potential candidates. (A-B) TIGAR siRNA knockdown decreases cell growth in OVCA420* cells. (A) Western blot analysis of TIGAR expression after siRNA transfection. β-Actin was used as a loading control. Cells were collected 72 hours after siRNA transfection before Western blotting. (B) SRB cell viability assay in OVCA420* cells with siRNA transfection. 48 hours after siRNA transfection, cells were treated with increasing concentrations of olaparib for 3 days before SRB assay. Data are shown as the raw data of fluorescent signal. Data are shown as mean with SEM. A representative of experiment with

triplicates is shown. **(C)** TIGAR amplification was correlated with a higher mRNA expression. **(D)** Short-term SRB cell viability assay indicates no change of sensitivity to olaparib after transfection with pooled siRNAs for select candidates identified from the CRISPR knockout screen. 48 hours after siRNA transfection, cells were treated with increasing concentrations of olaparib for 3 days before SRB assay. The vehicle-treated scr siRNA-transfected group was set at 100% cell survival. Data are shown as mean with SEM. Results are representatives from one experiment with triplicates. **(E)** Long-term colony formation assay also indicates no change in olaparib sensitivity after transfection with pooled siRNAs for each candidate. 48 hours after siRNA transfection, cells were treated for 3 days with increasing concentrations of olaparib and allowed to recover for 2 weeks before colony staining with SRB. Data are shown as mean with SEM. Results are representatives of experiments with duplicates.

Supplementary Figure 3.

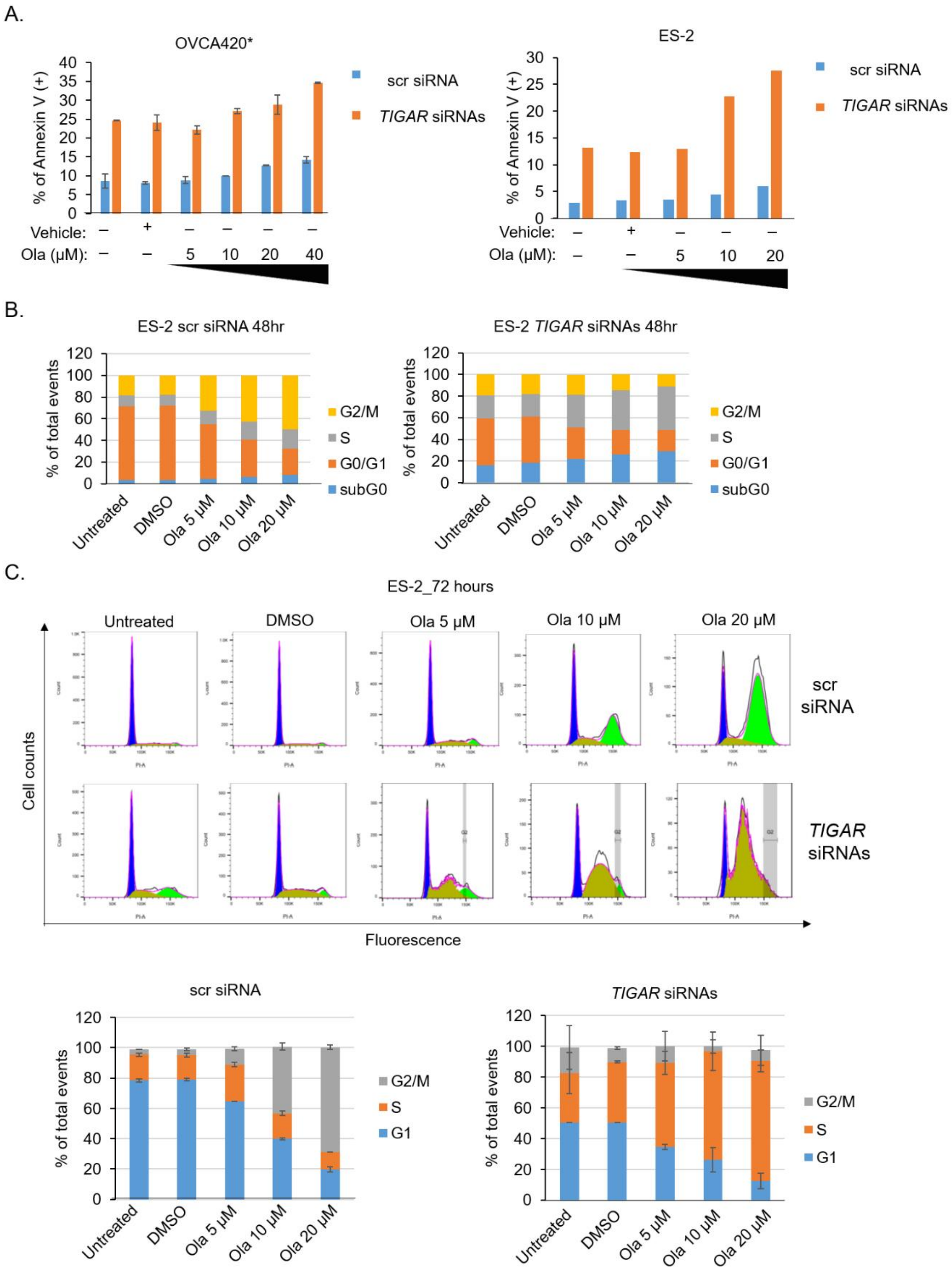

Supplementary Figure 3. Effects of TIGAR knockdown on apoptosis and cell cycle. (A) TIGAR knockdown results in an increase of apoptosis and the enhanced cytotoxicity for olaparib in OVCA420* and ES-2 cells. Cells were treated with vehicle or different concentrations of olaparib for 48 hours after TIGAR siRNAs transfection. Apoptosis analysis was performed by flow cytometry after Annexin V/PI staining. Annexin V-positive cells represent both early and late apoptotic cells. For OVCA420*, data are shown as mean with SD. Results represent an experiment with duplicates. For ES-2, the representative experiment was shown. **(B-C)** TIGAR knockdown increases S-phase fraction. Cell cycle analysis was performed in ES-2 cells treated with olaparib for 48 hours (B) or 72 hours (C) after siRNA transfection. Quantification of cell cycle distributions at 48 hours of olaparib treatment (B). The result is from one representative experiment. (C, upper panels) Representative cell cycle profiles analyzed in FlowJo. (C, lower panels) Quantification of cell cycle distributions at 72 hours of olaparib treatment.

Supplementary Figure 4.

A.

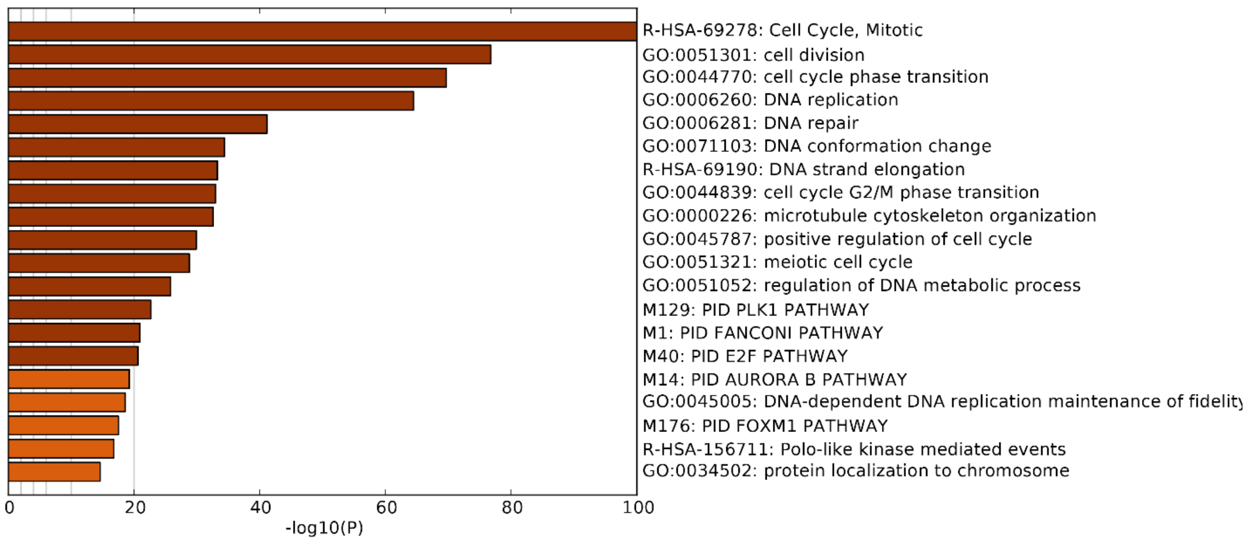

B.

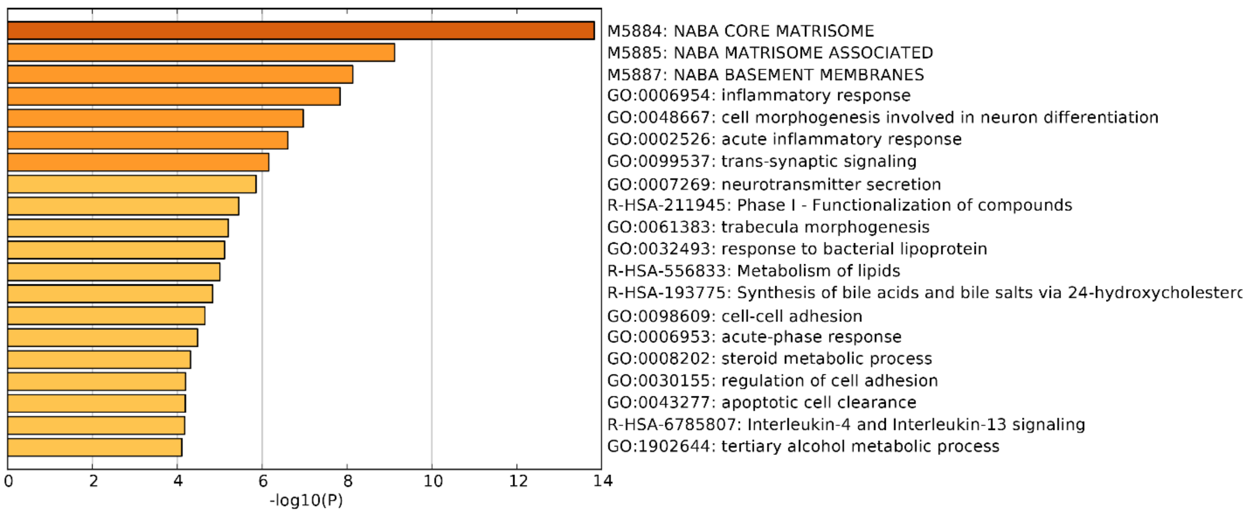

Supplementary Figure 4. RNA sequencing analysis after TIGAR knockdown. (A) Significantly downregulated genes (1108 genes by 2-fold at FDR≤0.001) were analyzed with Metascape bioinformatics web program. The analysis showed a significant enrichment of gene ontology term associated with the mitotic cell cycle, DNA repair, DNA strand elongation, the Fanconi Pathway and the FOXM1 pathway, suggesting that these processes and pathways are negatively affected by TIGAR KD. **(B)** Statistically enriched gene ontology terms that are associated with genes upregulated in TIGAR knockdown, including inflammatory response, metabolism of lipids, and apoptotic cell clearance.

Supplementary Figure 5.

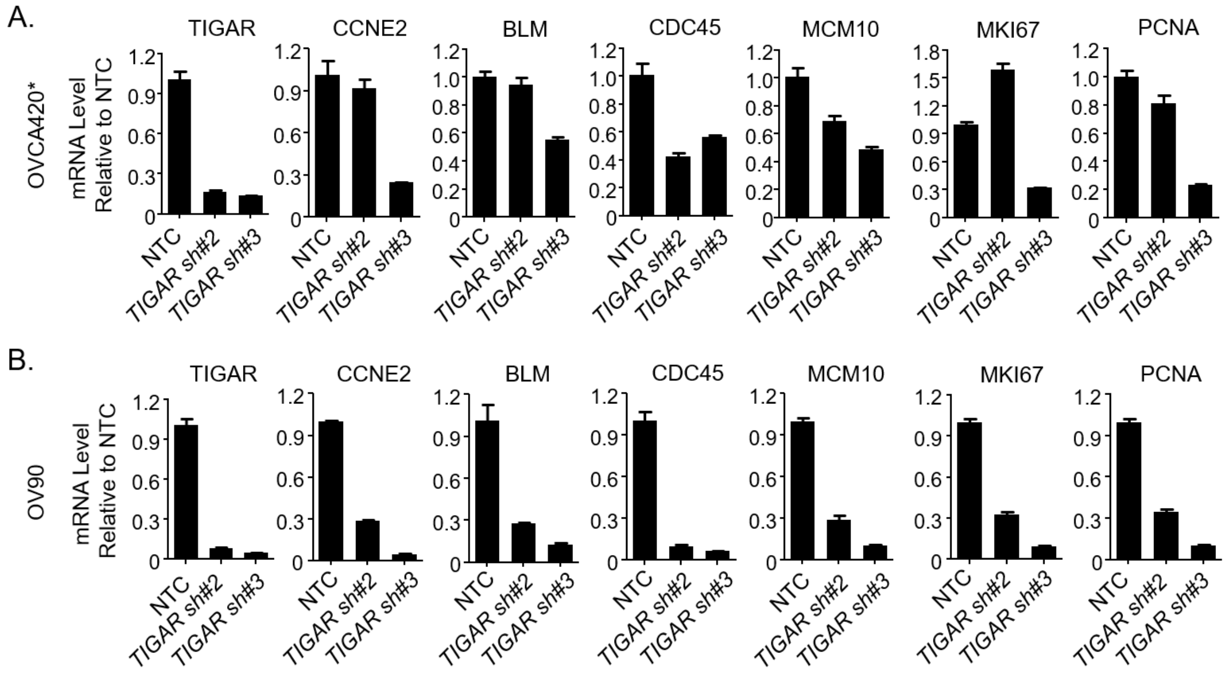

Supplementary Figure 5. Validation of gene expression for downregulated genes in OVCA420* (A) and OV90 (B) cells that were transduced with TIGAR shRNA. In general, the extent of downregulation of these candidate genes is bigger when the TIGAR is more efficiently downregulated with shRNA. Specifically, TIGAR sh#3 downregulated TIGAR more efficiently and was accompanied by greater downregulation of candidate genes.

Supplementary Figure 6.

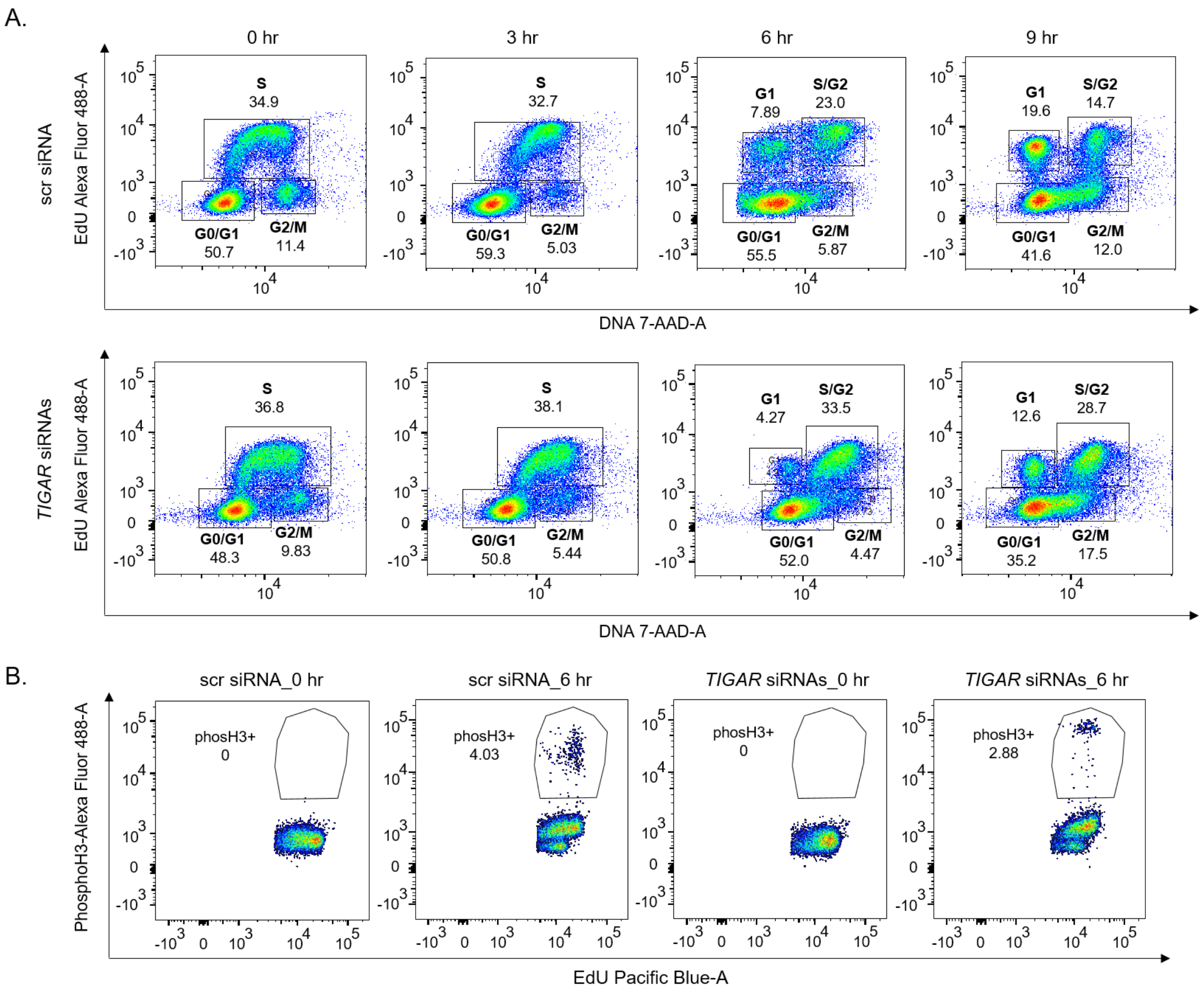

Supplementary Figure 6. TIGAR knockdown results in cell cycle progression delay. (A) Representative images showing profiles of cell distribution after EdU pulse labeling. **(B)** Representative images showing EdU positive mitotic cells using phospho-histone H3 as a marker. Both 0 hour and 6 hours after EdU pulse labeling were shown respectively.

Supplementary Figure 7

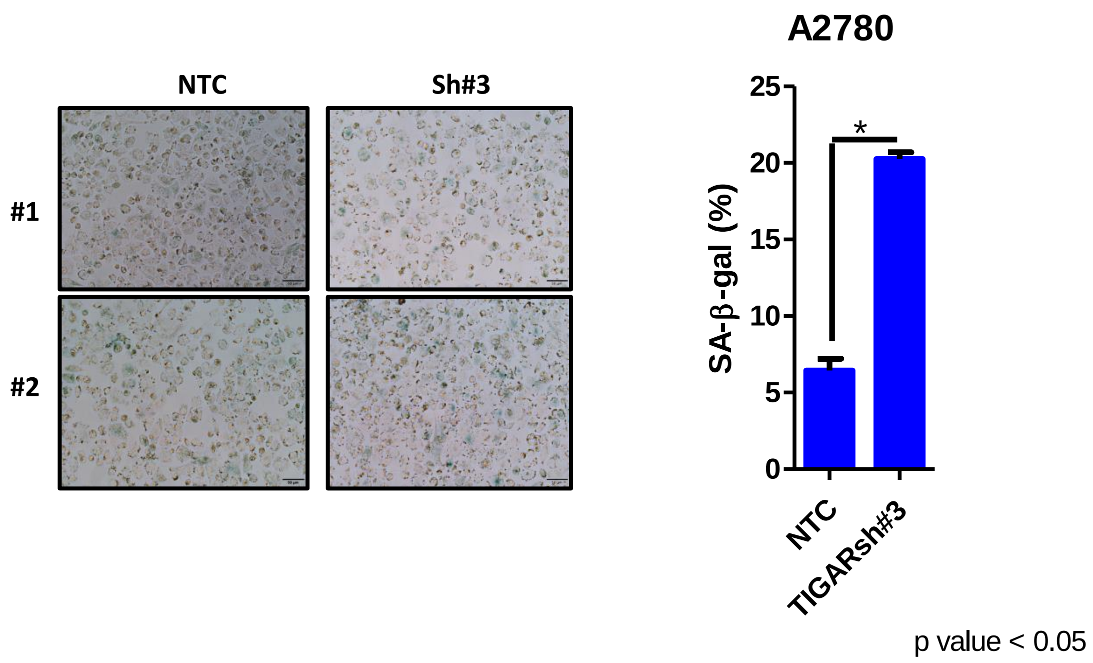

Supplementary Figure 7. The knockdown of TIGAR induces senescence. Senescence was determined by senescence-associated (SA)-β gal staining. Representative micrographs of duplicate samples (#1 and #2) from each group NTC (non-targeting shRNA control) and sh#3 (shRNA#3 targeting TIGAR) are shown. Image J was used to quantify β-gal stained cells, and the results were analyzed using GraphPad Prism (v. 5). * = $p < 0.05$.

Supplementary Figure 8.

A.

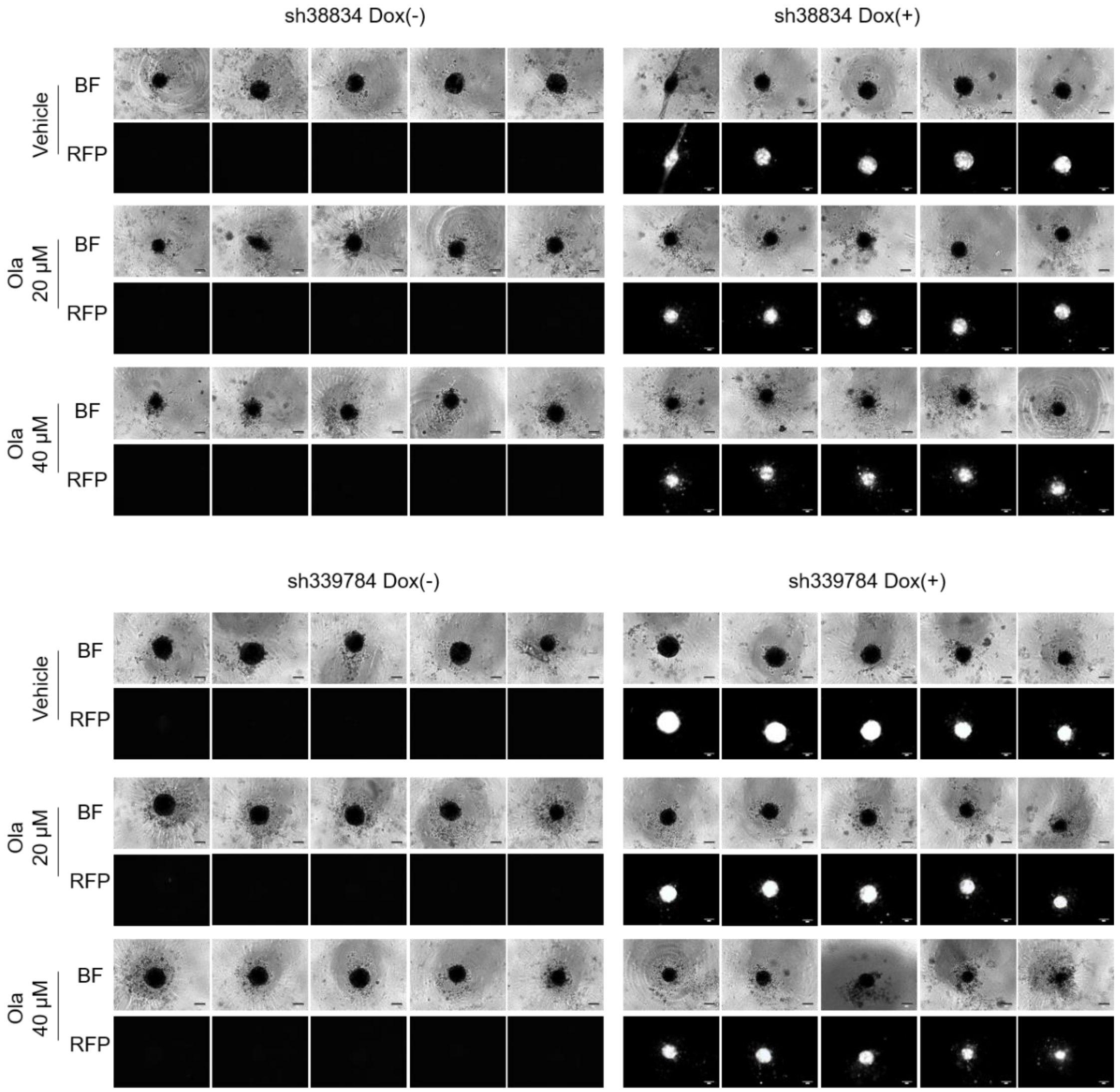

B.

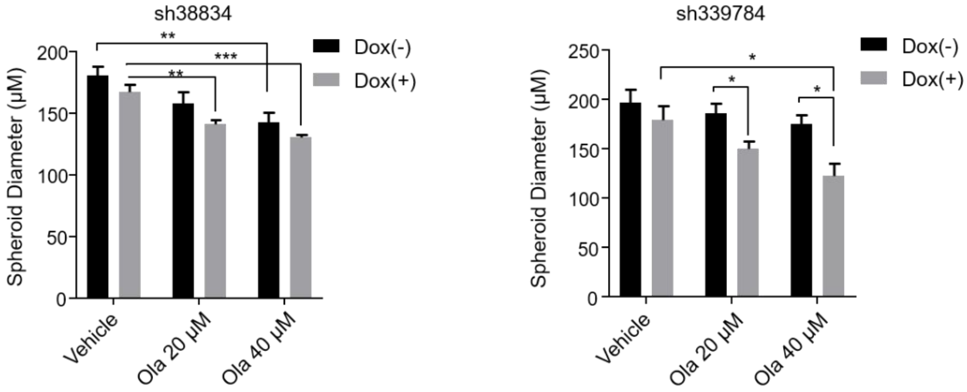

Supplementary Figure 8. Effects of TIGAR knockdown on the size of tumor spheroid treated with PARP inhibitor, olaparib. (A) Representative spheroid images with or without RFP (red fluorescent protein) expression. The absence of RFP in doxycycline (Dox)-deficient media indicates low levels of leakiness and a tight regulation of gene expression. Scale bar is 100 µm. OVCA420* cells stably transduced with inducible shRNAs were treated with 1 µg/mL doxycycline for 72 hours before seeding into Ultra low attachment spheroid 96 well plates at a plating density of 3000 cells per well. Cells were treated with vehicle, 20 or 40 µM olaparib for 10 days after spheroids formed. Doxycycline (1 µg/mL) was kept in the media throughout the experiment. **(B)** The spheroid size was quantified using Image J software from NIH. Quantification data from 5 spheroids of one representative experiment were used to generate the bar graph in Prism 6 software. Data are shown as mean ± SEM. Statistics was done with Student’s t-test. *=p<=0.05, **=p<=0.01, ***=p<=0.001.

Supplementary Figure 9. Uncropped immunoblots

Figure 1A

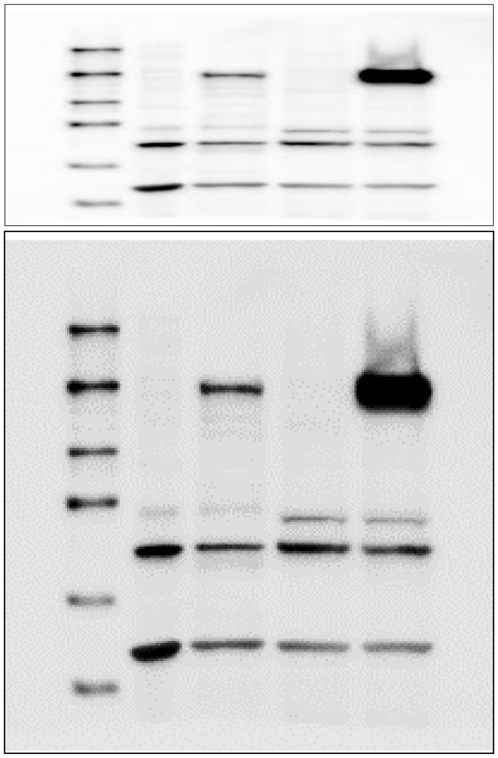

Figure 2F

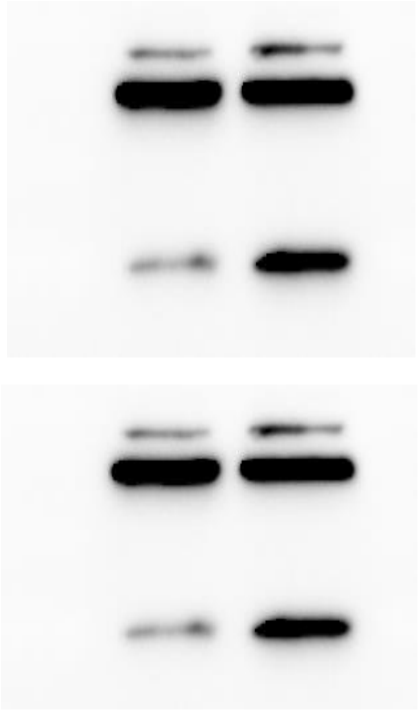

Figure 3B

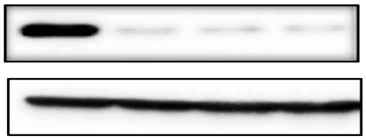

Figure 3F

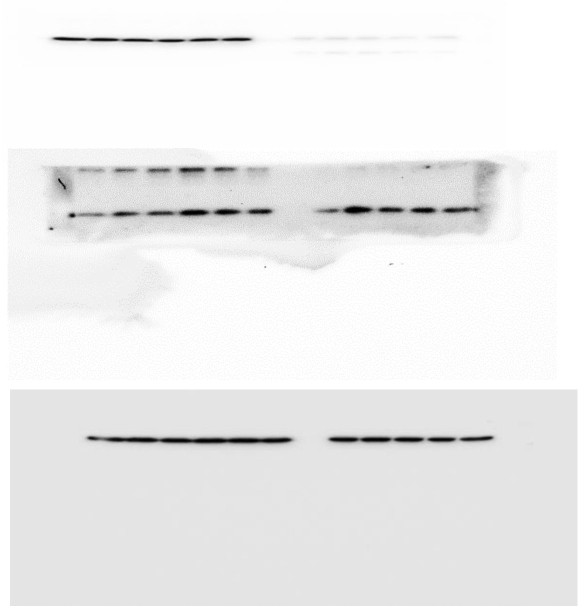

Figure 3D

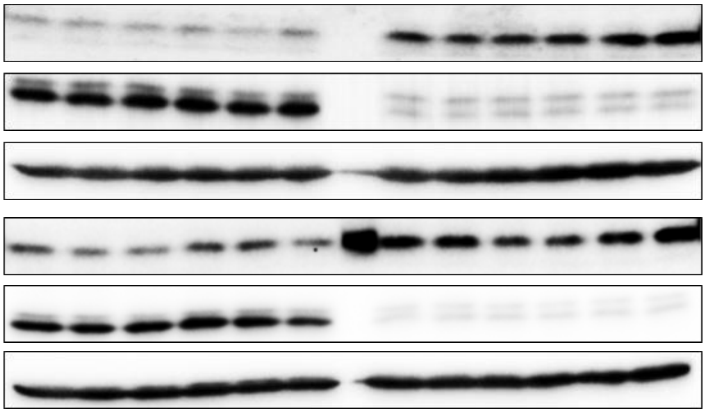

Figure 4A

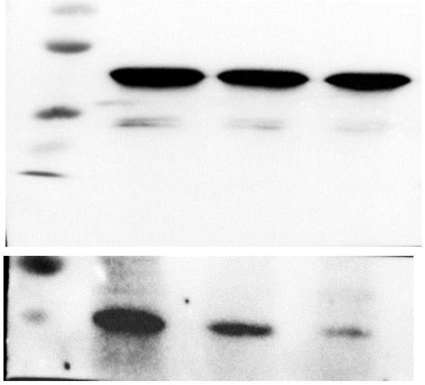

Figure 4A

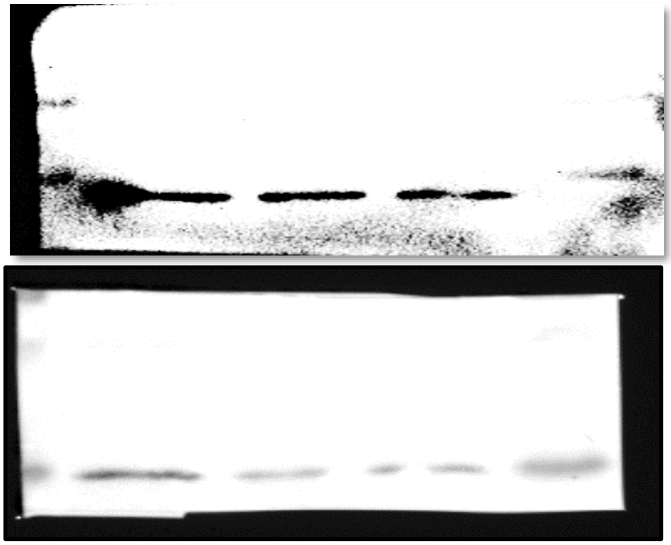

Figure 5D

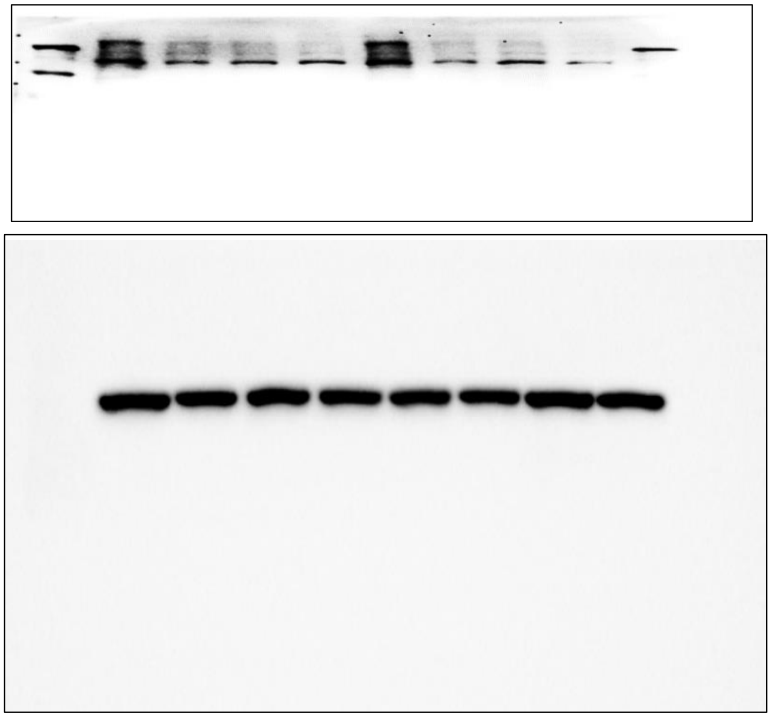

Figure 5F

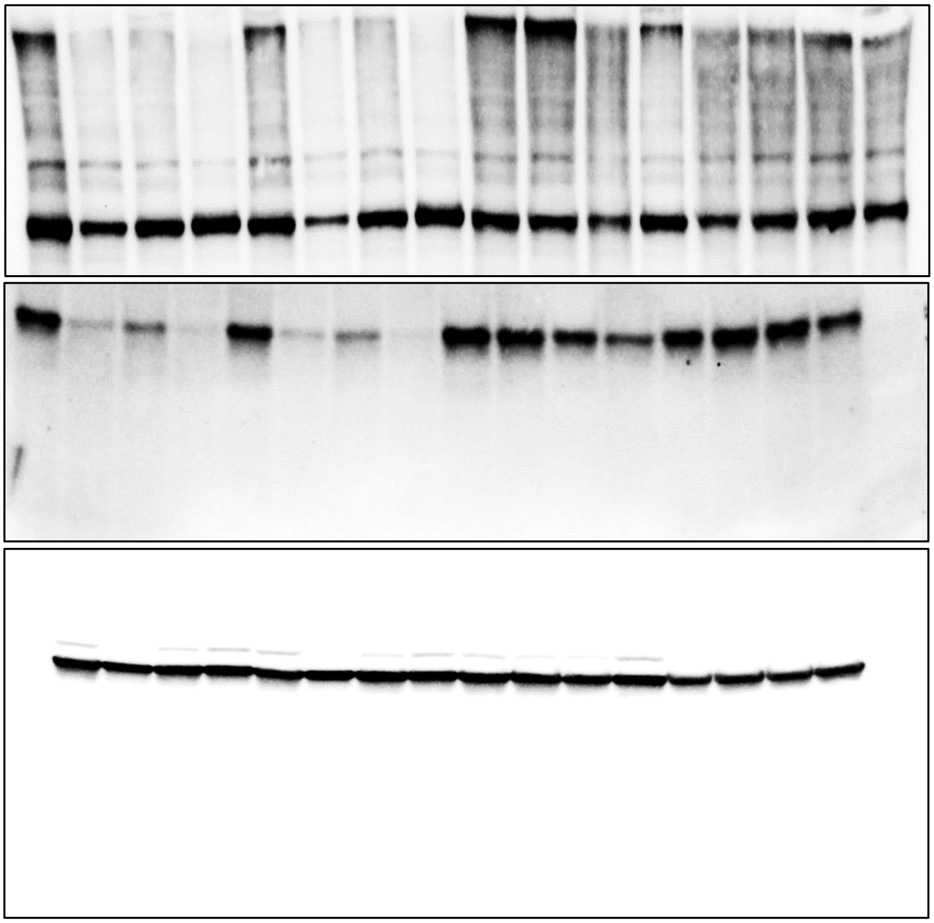

Figure 7B

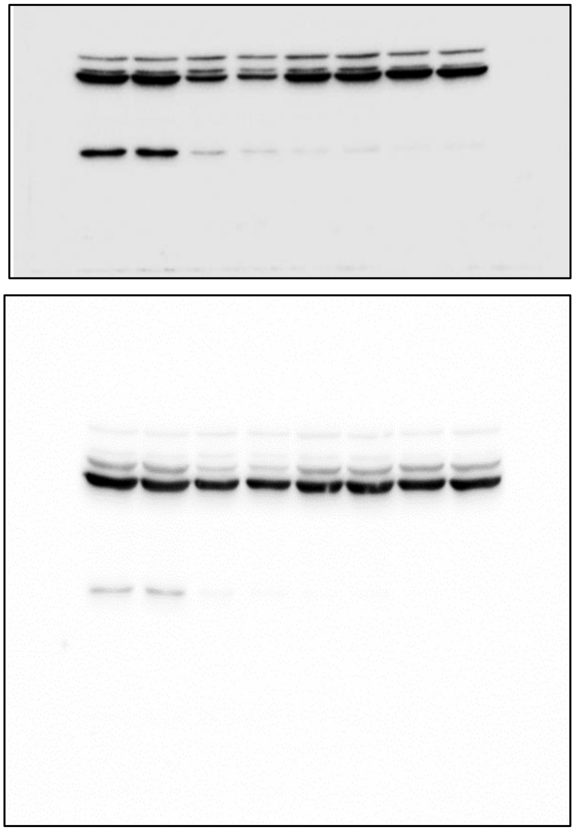

Figure 7C

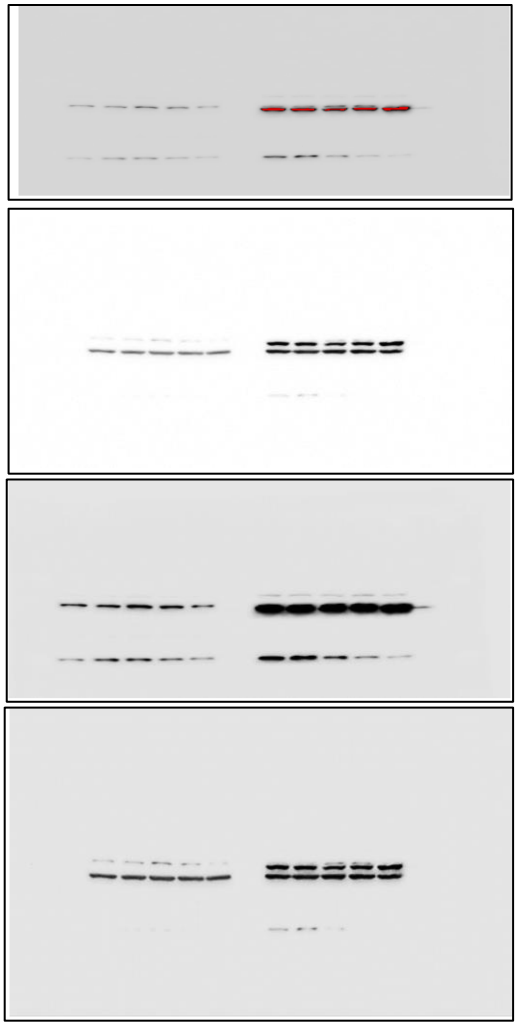

Figure 7E

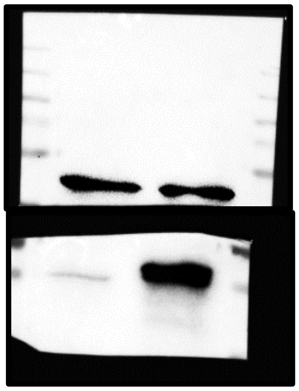

Figure 7F

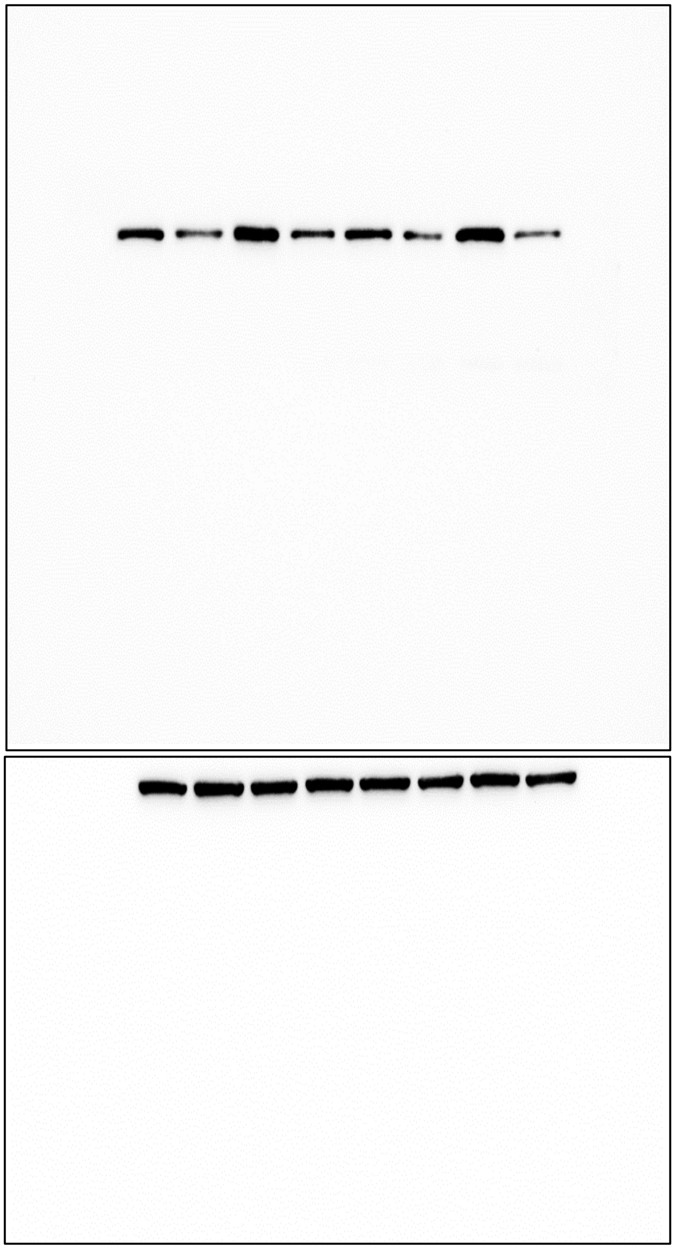

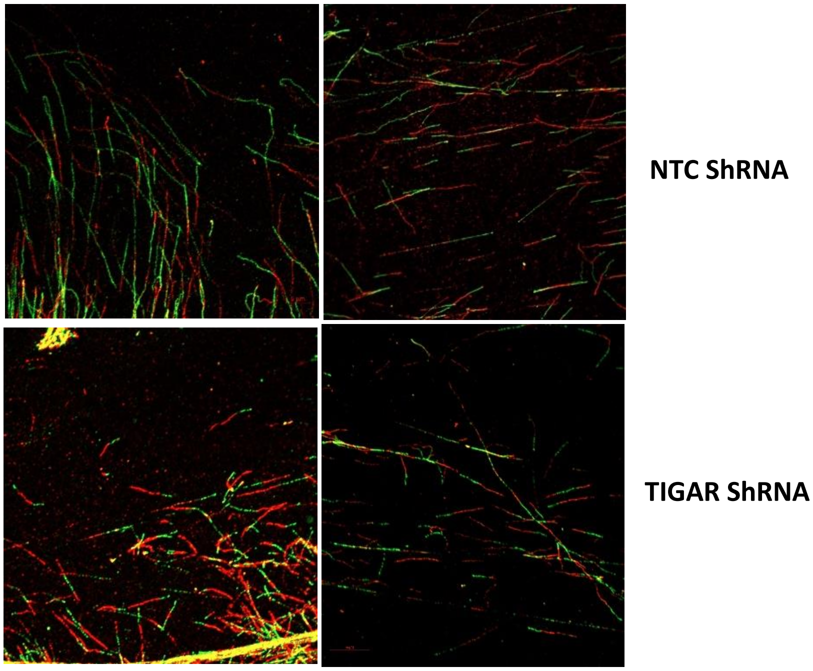

Supplementary Figure 10. (A) Scheme of pulse labeling with IdU and CldU for fiber combing assay and four major types of DNA fibers. Additional images of DNA fibers from OVCA420* cells with NTC (Non Targeting Control) shRNA or *TIGAR* shRNA were shown on the right.

Supplementary Table 1: DsiRNA sequences for candidate genes.

siRNA	Sense Strand	Antisense Strand
C12orf5 (TIGAR) siRNA #1	rGrGrArCrArArCrCrArUrArUrArG rArArUrUrArArCrUrUAT	rArUrArArGrUrUrArArUrUrCrUrA rUrArUrGrGrUrUrGrUrCrCrArU
C12ofr5 (TIGAR) siRNA #2	rGrCrArArArGrArUrArUrGrArCrG rGrUrArArArGrUrArUGA	rUrCrArUrArCrUrUrUrArCrCrGrU rCrArUrArUrCrUrUrUrGrCrArA
PARP1 siRNA #1	rCrCrArArArGrGrArArGrGrArArC rGrCrUrArArCrArArUTT	rArArArUrUrGrUrUrArGrCrGrUrU rCrCrUrUrCrCrUrUrUrGrGrUrC
PARP1 siRNA #2	rArGrUrArUrGrUrUrArArGrArArC rArCrUrCrArUrGrCrAAC	GrUrUrGrCrArUrGrArGrUrGrUrU rCrUrUrArArCrArUrArCrUrUrC
NME2 siRNA #1	rCrUrGrGrGrUrCrUrArUrGrArArUr ArArGrArGrGrUrGrGAC	rGrUrCrCrArCrCrUrCrUrUrArUrU rCrArUrArGrArCrCrCrArGrUrC
NME2 siRNA #2	rArGrArCrCrArArUrCrCrArGrCrA rGrArUrUrCrArArArGCC	rGrGrCrUrUrUrGrArArUrCrUrGrC rUrGrGrArUrUrGrGrUrCrUrCrC
OR5J2 siRNA #1	rGrGrArUrCrArUrGrGrArUrArG rGrUrGrGrArArUrArGrUTA	rUrArArCrUrArUrUrCrCrArCrCrU rArUrCrCrArUrGrArUrCrCrUrG
OR5J2 siRNA #2	rUrCrUrArUrGrUrUrCrUrArUrArCrGr CrUrArGrGrGrArUTC	rGrArArUrCrCrCrUrArGrCrGrUrA rUrArGrArArCrArUrArGrArArA
IL21 siRNA #1	rCrCrArCrCrCrArArArGrArArUrUrCrC rUrArGrArArArGAT	rArUrCrUrUrUrCrUrArGrGrArArU rUrCrUrUrUrGrGrGrUrGrGrUrU
IL21 siRNA #2	rGrCrArGrUrUrGrGrArCrArCrU rArUrGrUrUrArCrArUrACT	rArGrUrArUrGrUrArArCrArUrArGrU rGrUrCrCrArArCrUrGrCrArA
scr siRNA	rCrUrUrCrCrUrCrUrCrUrUrUrCrUrCrU rCrCrCrUrUrGrUGA	rUrCrArCrArArGrGrGrArGrArGrArA rArGrArGrArGrGrArArGrGrA

Supplementary Table 2. qRT-PCR primers

**Gene**	**Forward**	**Reverse**
C12orf5 (TIGAR)	5'GGAAGAGTGCCCTGTGTTTAC	5'AGTTGCTTGGAGATCCTTGG
PARP1	5'AGAGAAAAGGCGATGAGGTG	5'TTAGCTCGTCCTTGATGTTCC
NME2	5'CGAGCAGAAGGGATTGGC	5'TTCATGTACTTCACCAGCCC
OR5J2	5'CAGCATTTGTGTTTCGGAGTG	5'AGCAAGGGACTCACAATGG
IL21	5’ATCAAGCTCCCAAGGTCAAG	5’AGCTGACCACTCACAGTTTG
GGT6	5’CTGAGACACAGCCGGAAAG	5’CCTCCTCCACTTCCTCCCTC
NBPF9	5’TTGAATGAGCATCTCCAGGC	5’TCTTCGTCATTTTCTGGGCTG
PA2G4	5’TTGTGGTTGATGTAGCTCAGG	3’GCAACTTTGTTCCAGGCTTC
PIK3C2G	5’GCAAATTTACTGGCGTGGAC	5’GACTTACATCCCACACTCCTG
BRCA1	5’TAATGCTATGCAGAAAATCTTAGAG	5’TACTTTCTTGTAGGCTCCTTTTGG
CCNE2	5’CTGCCTTGTGCCATTTTACC	5’GTCTTCAGCTTCACTGGACTAG
BLM	5’TGCTCTTGCTTACCATGCTG	5’GAATCACAAATCGCACGTCC
ASF1B	5’CCTTTCCACAGCCCCTTC	5’AAATTCCTCACTCTCAGCCG
CDC45	5’GGTTCAAGCACAAGTTTCTGG	5’GTACAGCTTGTCCAGGTTACTC
ECSO2	5’CTGTGGGATAAGTAGAATCTGGG	5’GGTGTTGGGTCAGAAAATGC
MAD2L1	5’GACAGATCACAGCTACGGTG	5’GGCGGACTTCCTCAGAATTG
MCM10	5’AACCAGCCATCAAGTCCATC	5’TGGGCTCTCAACTTCACTTG
MKI67	5’AAAAGAATTGAACCTGCGGAAG	5’AGTCTTATTTTGGCGTCTGGAG
MYBL2	5’TGTGGATGAGGATGTGAAGC	5’TGAGGCTGGAAGAGTTTGAAG
PCNA	5’CCGAAACCAGCTAGACTTTCC	5’GATGAGGTCCTTGAGTGCC
TCF19	5’TGGCCTCATCTCTGGGATC	5’TCTCCATCACTCAATTCCAGC
UBE2C	5’TCTGGCGATAAAGGGATTTCTG	5’CTTGAGTTTCTCTGGGACCG
GAPDH	5’GAAACTGTGGCGTGATGGC	5’CACCACTGACACGTTGGCAG
18S rRNA	5’GCCCGAAGCGTTTACTTTGA	5’TCCATTATTCCTAGCTGCGGTATC
